# Neuroglycome alterations of hippocampus and prefrontal cortex of juvenile rats chronically exposed to glyphosate-based herbicide

**DOI:** 10.3389/fnins.2024.1442772

**Published:** 2024-08-21

**Authors:** Joy Solomon, Cristian D. Gutierrez-Reyes, Jesús Chávez-Reyes, Sherifdeen Onigbinde, Bruno A. Marichal-Cancino, Carlos H. López-Lariz, Mia Beck, Yehia Mechref

**Affiliations:** ^1^Department of Chemistry and Biochemistry, Texas Tech University, Lubbock, TX, United States; ^2^Department of Physiology and Pharmacology, Center of Basic Sciences, Universidad Autonoma de Aguascalientes, Aguascalientes, Mexico

**Keywords:** glyphosate-based herbicides, neurotoxicity, N-glycans, Hippocampus, prefrontal cortex, LC–MS/MS

## Abstract

**Introduction:**

Glyphosate-based herbicides (GBHs) have been shown to have significant neurotoxic effects, affecting both the structure and function of the brain, and potentially contributing to the development of neurodegenerative disorders. Despite the known importance of glycosylation in disease progression, the glycome profile of systems exposed to GBH has not been thoroughly investigated.

**Methods:**

In this study, we conducted a comprehensive glycomic profiling using LC-MS/MS, on the hippocampus and prefrontal cortex (PFC) of juvenile rats exposed to GBH orally, aiming to identify glyco-signature aberrations after herbicide exposure.

**Results:**

We observed changes in the glycome profile, particularly in fucosylated, high mannose, and sialofucosylated N-glycans, which may be triggered by GBH exposure. Moreover, we found major significant differences in the N-glycan profiles between the GBH-exposed group and the control group when analyzing each gender independently, in contrast to the analysis that included both genders. Notably, gender differences in the behavioral test of object recognition showed a decreased performance in female animals exposed to GBH compared to controls (*p* < 0.05), while normal behavior was recorded in GBH-exposed male rats (*p* > 0.05).

**Conclusion:**

These findings suggest that glycans may play a role in the neurotoxic effect caused by GBH. The result suggests that gender variation may influence the response to GBH exposure, with potential implications for disease progression and specifically the neurotoxic effects of GBHs. Understanding these gender-specific responses could enhance knowledge of the mechanisms underlying GBH-induced toxicity and its impact on brain health. Overall, our study represents the first detailed analysis of N-glycome profiles in the hippocampus and PFC of rats chronically exposed to GBH. The observed alterations in the expression of N-glycan structures suggest a potential neurotoxic effect associated with chronic GBH exposure, highlighting the importance of further research in this area.

## 1 Introduction

The discovery of Glyphosate, also known as *N*-(phosphomethyl)glycine, dates back to 1950 when a Swiss chemist, Dr. Henri Martin, first identified it. Not until 1970 was it synthesized and tested as a herbicide by Franz et al. (1997). Glyphosate formulations have since grown to dominate the herbicide market (Benbrook, 2016; Duke, 2018; Kanissery et al., 2019), with a wide range of efficacy. In 1974, it received official approval for usage in the United States (Myers et al., 2016). Its main application was in the agricultural sector, where farmers utilized it to eradicate weeds and control their growth in non-crop regions and pastures (Simonetti et al., 2015). The mechanism of action is highly effective; it works by blocking the activity of the enzyme known as 5-enol-pyruvyl-shikimate-3-phosphate synthase (EPSPS) (Jaworski, 1972; Steinrücken and Amrhein, 1980; Rubin et al., 1982; Kataoka et al., 1996), which catalyzes the sixth step in the shikimic acid pathway (Cole, 1985). Inhibiting the enzyme activity hinders the production of essential plant metabolites, including aromatic amino acid hormones (Amrhein et al., 1980). Glyphosate serves as a key ingredient in various formulations referred to as Glyphosate-Based Herbicides (GBHs) (Guyton et al., 2015). These herbicides are primarily used to hinder the growth of weeds and some types of perennial weed plants in industrial and residential environments (Dill et al., 2010). GBHs are extensively utilized on a wide variety of food crops. Yet, the application of glyphosate on these crops can lead to the presence of glyphosate and its main metabolite aminomethylphosphonic acid as residues in the crops upon harvest. Its extended usage has resulted in the presence of measurable levels of glyphosate residues in water (Coupe et al., 2012), soil (Silva et al., 2018), food (Simonetti et al., 2015), human serum (Yoshioka et al., 2011), urine (Niemann et al., 2015), breast milk, and more (Steinborn et al., 2016). Glyphosate was initially regarded as benign with low mammalian toxicity (Baylis, 2000), due to the absence of the target of GBH (the shikimate pathway) in humans. While regulatory agencies in the United States and Europe classify glyphosate as a chemical with minimal carcinogenic potential, the International Agency for Research on Cancer (IARC) categorizes it as a probable carcinogen. This classification is based on its capacity to induce DNA damage and oxidative stress, which are considered key factors in determining carcinogenicity (Guyton et al., 2015; Fogliatto et al., 2020; Kudsk and Mathiassen, 2020). Exposure to GBH has, however, been implicated in kidney disease (Jayasumana et al., 2014), birth defects in humans, endocrine disruption (Walsh et al., 2000; Ingaramo et al., 2020), cancer (Thongprakaisang et al., 2013; Guyton et al., 2015), and Parkinson’s disease (Wang et al., 2011). Studies have also shown that GBH exposure plays a crucial role in neurological impairments (Ait-Bali et al., 2020; Cattani et al., 2021) and neurotoxicity (Roy et al., 2016; Cattani et al., 2017; Chávez-Reyes et al., 2024a,b).

The exact molecular and cellular mechanisms by which glyphosate produces its deleterious effects on mammals and other animals remain mostly undetermined. However, it was reported *in vitro* that an important toxic action of glyphosate may be linked with generation of reactive oxygen species (ROS) and alteration to the mitochondrial functions (Strilbyska et al., 2022). Mitochondrial dysfunction and ROS production was also observed in cardiomyoblasts exposed to glyphosate with reduction on cells viability (Arrigo et al., 2023). Moreover, in mature rat testicular cells, high concentrations of glyphosate produced necrosis, apoptosis and endocrine affectations (Clair et al., 2012). Other molecular actions include decrease in the acetylcholinesterase activity in rat brain areas such as the hippocampus and prefrontal cortex (Chávez-Reyes et al., 2024b). Interestingly, glyphosate may reach the brain and increases pro-inflammatory cytokine such as TNFα (Winstone et al., 2022). We reported previously that four important metabolites (paraxanthine, epinephrine, L-(+)-arginine, and D-arginine) presented important changes after exposition to glyphosate (Daramola et al., 2024). Nevertheless, the biological consequences of the mentioned molecular, cellular and metabolomic effects remains to be better understood.

Posttranslational modifications (PTMs) are crucial for controlling various cellular functions in living organisms (Nørregaard Jensen, 2004). There are multiple categories of PTMs, such as acylation, acetylation, phosphorylation, oxidation, and glycosylation. Glycosylation is considered the most common post translational modification (Hart and Copeland, 2010), with more than 50% of the mammalian proteins glycosylated (Wong, 2005). It is an enzymatic alteration of proteins and lipids by sugars known as glycans (Varki et al., 2015). Protein glycosylation can be classified into two distinct types, namely *N*-glycans and *O*-glycans. *N*-glycans are attached to an asparagine amino acid residue with a motif Asn-X-Ser/Thr (where X can be any amino acid except Pro), while *O*-glycans are commonly linked to either serine or threonine amino acid residues, in the absence of a motif. The *N*-glycans share a common core structure of GlcNAc_2_Man_3_, whereas *O*-glycans display various core configurations (Veillon et al., 2017, 2018). Omics studies have greatly expanded our comprehension of diseases (Ressom et al., 2016), several of which have been found to be associated with changes observed in research areas such as proteomics (Sanni et al., 2023), glycoproteomics (Mechref et al., 2023), glycomics (Onigbinde et al., 2023; Reyes et al., 2023), and metabolomics (Daramola et al., 2024). In addition to the primary emphasis on protein biomarkers, glycomics investigations have demonstrated significant physiological importance. Their clinical relevance and use in support of accurate diagnostics for numerous conditions are increasingly recognized, even leading to the development of novel medical fields such as “pathomics” (Bülow et al., 2023). Glycosylation has gained attention over time due to the roles it plays in many biological functions such as cell–cell recognition (Ohtsubo and Marth, 2006), cell adhesion (Phillips et al., 1990; Oyama et al., 2018; Singh et al., 2018), protein stability (Solá and Griebenow, 2009), and immune cell trafficking (Sperandio et al., 2009). Aberrant glycosylation has been implicated in diseases such as diabetes (Bermingham et al., 2018), Parkinson’s disease (Russell et al., 2017), Alzheimer’s Disease (Cho et al., 2019), and various cancers (Hakomori, 1996; Dennis et al., 1999; Mechref et al., 2012; Onigbinde et al., 2024). The connections between disease development, progression, and changes in glycosylation have prompted numerous research endeavors aimed at potentially using these variations to provide dependable diagnostic information. Moreover, the capacity to identify alterations in glycans with great accuracy and sensitivity could have significant ramifications for predicting disease outcomes (Mechref et al., 2012).

In this study, we present the profiling of permethylated *N*-glycans from brain tissues of rats exposed to GBH, using highly sensitive liquid chromatography tandem mass spectrometry (LC–MS/MS) and parallel reaction monitoring (LC-PRM-MS) for their validation. Mass spectrometry is widely employed for analyzing glycans because of its exceptional sensitivity and ability to provide data rich in structural information, resolution, and mass accuracy (Dell and Morris, 2001; Mechref and Novotny, 2002; Zhou et al., 2017; Dong et al., 2018). The integration of liquid chromatography with mass spectrometry (LC–MS/MS) has gained significant popularity for the characterization and quantification of glycans, thereby revealing their heterogeneity and biological functions (Veillon et al., 2017; Dong et al., 2018; Peng et al., 2022). Glycans are increasingly considered as potential biomarkers for studying disease and its progression. Hence, the differences in expression of *N*-glycans were investigated herein to provide insight into the various changes that occur in the hippocampus and PFC after exposure to GBH. This information facilitates the identification of *N*-glycans that exhibit significant changes in expression after exposure to GBH. Following this research, it will be necessary to conduct mechanistic studies to ascertain the distinct functions of the *N*-glycans and clarify the fundamental mechanisms associated with chronic exposure to GBH.

## 2 Materials and methods

### 2.1 Chemicals and reagents

Acetic acid, ammonium bicarbonate (ABC), borane–ammonia, formic acid (FA), sodium deoxycholate (SDC), di-methyl sulfoxide (DMSO), iodomethane, and sodium hydroxide (NaOH) beads were purchased from Sigma-Aldrich (St. Louis, MO, United States). PNGase F enzyme was purchased from New England Biolabs (Ipswich, MA, United States). High-performance liquid chromatography (HPLC) grade methanol (MeOH), water, and acetonitrile (ACN) were obtained from Fisher Scientific (Fair Lawn, New Jersey, United States). Molecular biology grade zirconium beads were obtained from OPS Diagnostics (LLC, Lebanon, NJ). Micro-columns were acquired from HA (Holliston, MA, United States), and the Isolute^®^ C18 (EC) cartridges were acquired from Biotage (Charlotte, NC, United States). The GBH used for this research was the Rival^®^ herbicide from Monsanto (St. Louis, MO, United States) with a concentration of 680 g/kg of glyphosate “N-(phosphonomethyl)glycine.” The granulate product was used to prepare GBH solutions with a concentration of 100 mg/mL. The solution was prepared fresh every day using injectable water as solvent. Pentobarbital sodium was purchased from PETS Pharma Ltd. (Ags., Mexico).

### 2.2 Animal study

**Figure 1:**
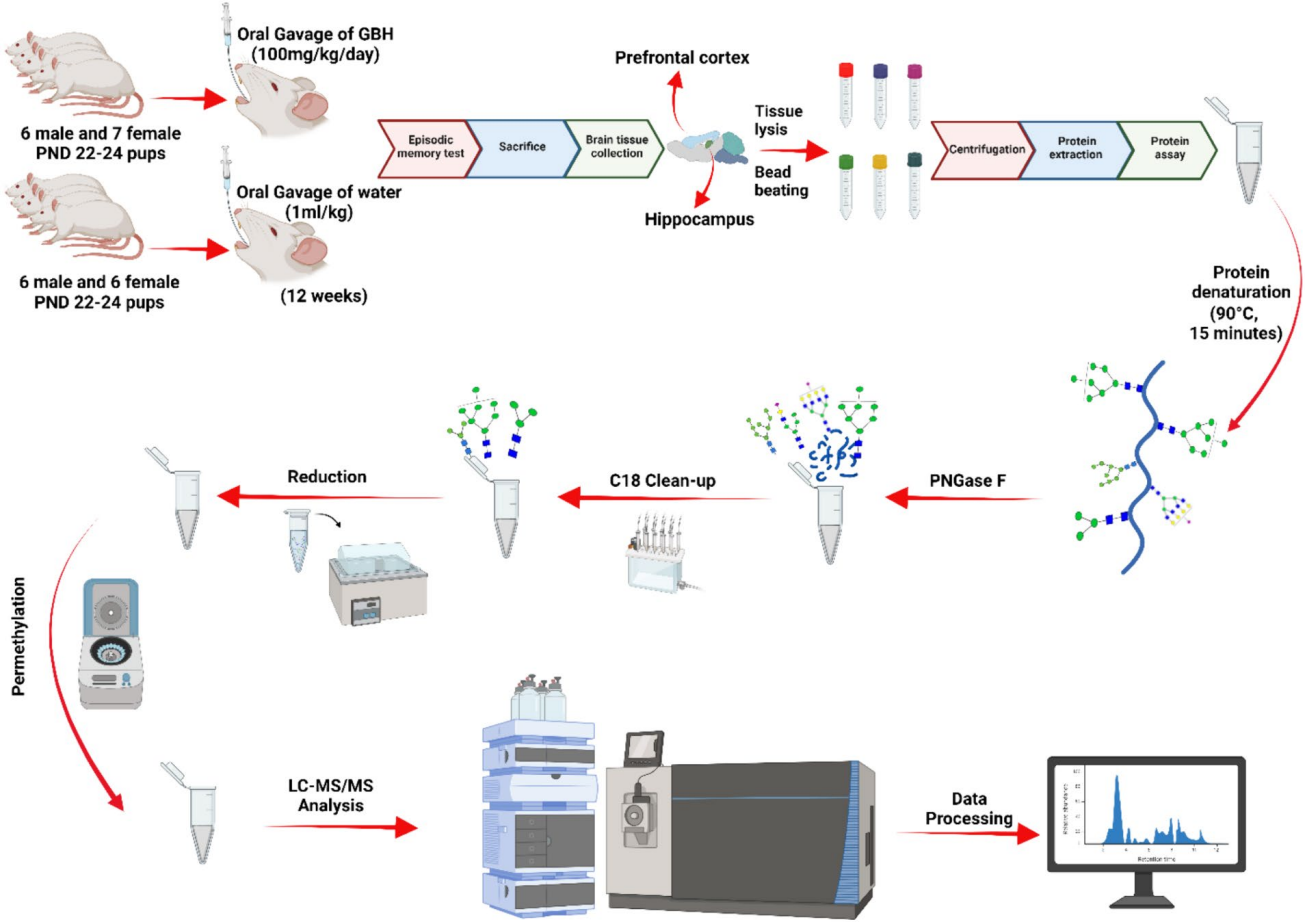
*N*-glycomics workflow. The data underwent normalization through analyzing the overall abundance of glycan expression across the samples. Afterwards, a parallel reaction monitoring (PRM) analysis was conducted to validate the significant *N*-glycans. A five-digit *N*-glycan nomenclature was used in the following order: *N*-acetylglucosamine, hexose, fucose, *N*-acetylneuraminic acid, *N*-glycolylneuraminic acid (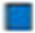 HexNAc, 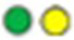 Hex, 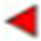 Fuc, 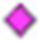 NeuAc, 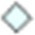 NeuGc).

For this study, twenty-five Sprague Dawley (SD) rats were utilized: 12 males (6 control and 6 GBH-exposed) and 13 females (6 control and 7 GBH-exposed). They were obtained from the institutional vivarium of the Autonomous University of Aguascalientes, at postnatal days 22–24, and were housed in groups of three or four rats per cage. The experimental procedures adhered to the Mexican Guidelines for Animal Care NOM-062-ZOO-1999 and the National Research Council Guide for the Care and Use of Laboratory Animals (Bayne, 1996). The rats were housed in a controlled environment with a 12 h light/dark cycle (the light was on at 7:00 h). The temperature was maintained between 20–22°C, and the humidity was regulated at 45–55%. The rats had unrestricted access to food and water. A total of twelve SD rats, consisting of six males and six females, were allocated to the control group. The control group received daily oral gavages of water, with a dosage of 1 mL per kg of body weight, for a duration of 12 weeks. The GBH-treated group consisted of 13 SD rats, comprising 6 males and 7 females, who were administered GBH orally at a dosage of 100 mg of glyphosate/kg/day for a duration of 12 weeks. The administration started a couple days after the rats were received from vivarium. They were administered 7 days a week. Since the main goal of this protocol was to evaluate the neuroglycome alterations due to chronic exposure to GBH, the dosage was determined according to previous reports where oral exposure to 100 mg/kg of GBH induced a decrease in the activity of AChE from brain tissues (Gallegos et al., 2018). In addition, similar concentration of GBH administrated sub chronically via gavage induces behavior and memory impairments (Bali et al., 2019; Bicca et al., 2021). After the 12 weeks exposure protocol, the novel object recognition (NOR) test was conducted on the rats. Lastly, the rats were sedated with pentobarbital sodium and then euthanized with an overdose of pentobarbital sodium via intraperitoneal injection. Immediately after euthanasia, the whole hippocampus and PFC of the rat were extracted and frozen until use. The brain tissue samples underwent LC–MS/MS glycomics analysis. Figure 1 depicts the sequential process of the investigation.

### 2.3 Episodic memory evaluation by novel object recognition test

The NOR test, which is an episodic learning and memory test for rodents (Leger et al., 2013; Panoz-Brown et al., 2016), was developed in 3 days with minor modifications. The object recognition test is widely regarded as a successful approach for assessing discriminative-declarative memory in rodents. This memory predominantly relies on the functioning of the hippocampus and medial prefrontal cortex (Drayson et al., 2023). Various approaches of NOR have been documented, including one that involves three sessions divided into habituation, training, and testing (Antunes and Biala, 2012). This approach does not require extensive training schedules because rodents innately have a preference for novelty (Lueptow, 2017). This methodology employed previously (Bevins and Besheer, 2006; Lueptow, 2017), omits the short-term memory analysis and focuses only on long-term discriminative memory analysis giving a 24 h memory consolidation period between training and testing sessions to get a more comparable results through discriminative indexes. Briefly, the habituation session to NOR (day 1) consisted of one session (for 5 min) of free exploration into the open field (OF) apparatus, as described previously (Goulart et al., 2010; Lueptow, 2017). The OF apparatus consisted in an open field arena: square platform 60 cm × 60 cm × 30 cm. Experiments were performed in an isolated room with white light intensity of 25-W and sessions started at 12:00 h and were videorecorded; data collection was manually analyzed by a blind observer. Then, a familiarization phase was developed on day 2. The familiarization phase was performed as described by Lueptow (2017) in which two identical objects were placed at opposite quadrants. Animals had to explore each object at a minimum time of 20s in a 5 min session. Animals that failed to explore any of the objects for less than 20s would have been discarded for the NOR-test. No animals were discarded in this study as all reached the minimum time in the familiarization phase. Lastly, on day 3, a test of preference for a particular object was performance. In the test of preference, one of the known objects were randomly replaced by a new one with a different shape and color but similar size. The preference test session lasted 5 min. The objects and OF arena were washed with 70% ethanol before each session. Object exploration was defined as touching, sniffing, and/or nibbling the objects, and the time was used to calculate the Discrimination Index (DI) with the following formula:


Discrimination index DI=novel objectexploration time−familiar objectexploration timetotal exploration time of two objects×100

### 2.4 Tissue lysis and protein extraction

Aliquots of 100 μL of 50 mM ABC buffer (pH = 7.5) were added to the brain tissue samples (~1 mg). Next, they were carefully transferred into an empty 2.0 mL microcentrifuge tube (conical, with screw cap), already filled with 100 mg of zirconium beads (400 μm molecular biology grade zirconium beads—OPS Diagnostics, LLC, Lebanon, NJ). Then, 100 μL of 5% aqueous SDC solution was added to the microtubes containing the brain tissue samples. The sample was homogenized using BeadBug microtubes (Benchmark Scientific, Edison, NJ) at 4,000 rpm (1,541 × g), 4°C, for 30 s. This process was repeated five times with a 30 s break between each cycle. The lysate was sonicated in ice for 1 h. The supernatants were then removed. Formic acid was added to the samples to make its concentration 1%. The samples were centrifuged at 1,000 rpm for 1 min, then 14,800 rpm for 10 min. The supernatants were collected. The protein concentration from the prepared tissue lysates were determined using a bicinchoninic acid (BCA) protein assay kit (Thermo Sci.).

### 2.5 N-glycan release and purification

After the protein assay, the equivalent of 20 μg of protein was diluted to 50 μL with 50 mM ABC buffer and subjected to denaturization at 90°C for 15 min. Then, 1,000 U of PNGase F were added to the samples and incubated at 37°C for 18 h. The digested samples were dried and then reconstituted in 300 μL of 5% acetic acid. Simultaneously, the SPE C18 cartridges were conditioned with 3 mL of MeOH and equilibrated with 3 mL of an aqueous solution containing 5% acetic acid. The reconstituted samples were placed into the cartridges and rinsed with 300 μL of 5% acetic acid, repeated three times. The liquid that passed through was collected in 1.5 mL tubes and dried using a SpeedVac concentrator.

### 2.6 Reduction and permethylation of N-glycans

The released *N*-glycans were subjected to reduction with a method adopted from Palmigiano et al. (2016). The reducing reagent borane-ammonia complex was dissolved in HPLC water to a final concentration of 10 mg/mL. Then, 10 μL of freshly prepared reduction solution was added to each sample tube. They were incubated at 60°C for 1 h. The formed borate was removed from the samples by adding 1,000 μL of MeOH at least five times, until the formed methyl borate was completely dried in the vacuum concentrator. The reduced *N*-glycans were subjected to solid-phase permethylation (Kang et al., 2008; Zhou et al., 2017). Briefly, the samples were reconstituted in 30 μL of DMSO, 1.2 μL of water, and 20 μL of iodomethane. The micro-spin column was filled with NaOH beads (suspended in DMSO) and spun down at 1800 rpm for 2 min. Next, the column was washed with 200 μL of DMSO, and centrifuged at the same speed. The sample solution was introduced to the micro-spin column. The column was then kept in darkness at room temperature for 25 min. Next, 20 μL of iodomethane was added and incubated for an additional 15 min. After the second period of incubation, the columns were centrifuged at 1800 rpm for 2 min to collect eluent. Finally, 30 μL of ACN was added to the column for the second elution. The permethylated *N*-glycans were isolated using centrifugation at 1800 rpm, dried, and then reconstituted in an aqueous solution comprising 20% acetonitrile and 0.1% formic acid before being subjected to LC–MS analysis.

### 2.7 LC–MS/MS conditions

LC-ESI-MS/MS was performed on an UltiMate 3,000 nanoUHPLC system (Thermo Sci., San Jose, CA, United States) coupled with an Orbitrap Fusion Lumos Tribrid mass spectrometer (Thermo Sci., San Jose, CA, United States). The instrument was equipped with a nanoESI source, and two microliters (equivalent to 1 μg) of the permethylated *N*-glycan sample was introduced into the LC–MS system. The samples were online-purified to remove any remaining salts and impurities on C18 particles using a C18 trap Acclaim PepMap 100, 75 μm × 2 mm, 3 μm, 100 Å, Thermo Sci. The trapped samples were then separated using a C18 capillary column Acclaim PepMap 100, 75 μm × 150 mm, 2 μm, 100 Å, Thermo Sci. A chromatographic gradient with a column temperature of 55°C was used. The separation was achieved at a 0.35 μL/min flow rate using mobile phase solvents A (comprised of 98% water, 2% ACN with 0.1% FA) and B (comprised of 98% ACN with 0.1% FA). The chromatographic gradient was as follows: 0–10 min constant 20% solvent B; 10–11 min 20–42% solvent B; 11–48 min 42–55% solvent B; 48–49 min 55–90% solvent B; 49–54 min constant 90% solvent B; 54–55 min 90–20% solvent B; and 55–60 min constant 20% solvent B. The Orbitrap Fusion Lumos Tribrid mass spectrometer was operated in positive mode. The spray voltage was set at 1.6 KV with a capillary temperature of 305°C. This event was a full MS scan of 400–2000 m/z at a mass resolution of 120 K. The MS/MS scans were generated using a Data Dependent Acquisition (DDA), where the 20 ions with the highest intensity were selected for CID fragmentation. A collision energy (NCE) of 35% and activation time of 10 ms was set.

### 2.8 Parallel reaction monitoring LC–MS condition

A targeted PRM approach was employed to validate the differentially expressed *N*-glycans across the studied cohorts. A pooled sample was run on the UltiMate 3,000 nanoUHPLC system (Thermo Sci., San Jose, CA) coupled with an Orbitrap Fusion Lumos Tribrid mass spectrometer (Thermo Sci., San Jose, CA). The pooled sample was prepared and subjected to full scan analysis, then the results were used to generate the PRM transition list containing the *N*-glycan information: retention time, m/z, charge, and optimal collision energy for the precursor. The transition list was incorporated into the instrument method of the analytical system described in Section 2.7 and used to analyze the samples in PRM mode. A list of *N*-glycans validated by LC-PRM-MS, including precursor m/z, transition fragment ions, fold change (FC), and log2FC for the full scan and PRM validation, is shown in Supplementary Table S1. The PRM data was processed and quantified using Skyline^®^ software version 21.2.0.536 and the normalized data was collected and subjected to statistical analysis.

### 2.9 Data analysis

For the *N*-glycomics analysis, the raw data was revised using Xcalibur 4.2 (Thermo Sci.) software confirming retention time, MS, and MS/MS spectra. The total intensity of all *N*-glycan species was added together to represent the abundance of each *N*-glycan. After the absolute abundance was determined, the normalization process involved calculating the relative abundance of each *N*-glycan. This was accomplished by dividing the abundance of each individual *N*-glycan by the total abundance of all *N*-glycans. Ultimately, the relative quantitation results were compared and then subjected to principal component analysis (PCA). Statistical analyses were performed using the Mann Whitney U test. The *N*-glycans with statistical significance (*p* value <0.05) were further investigated using box graphs and ROC curves created with GraphPad Prism and SPSS software, respectively.

## 3 Results

### 3.1 Analytical workflow

**Figure 2:**
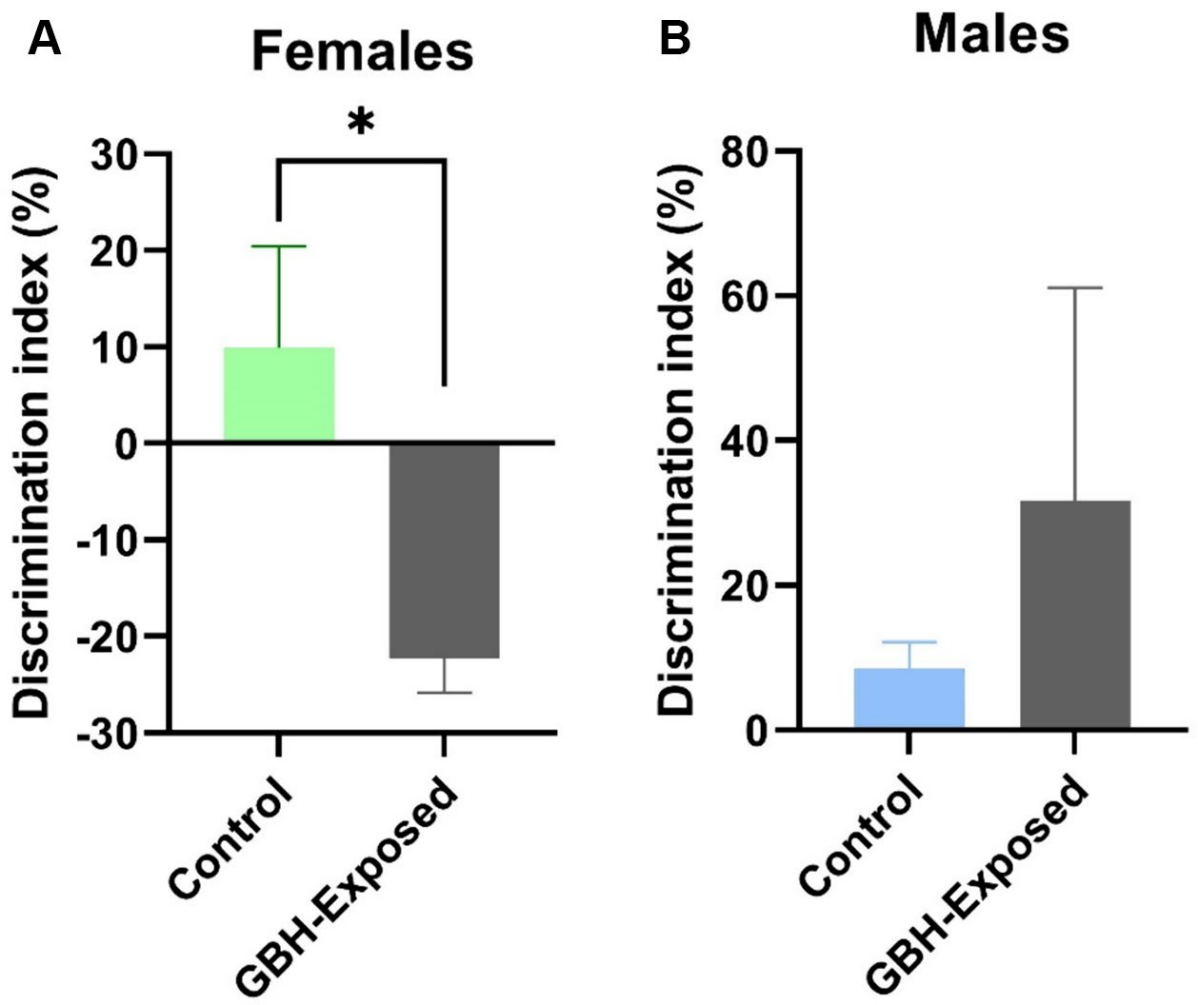
Effect of GBH on episodic memory in the novel object recognition (NOR) test. Discrimination index in the NOR test of **(A)** female and **(B)** male rats exposed during 12 weeks to GBH (*n* = 6 males/females in the control group; and 6 males and 7 females in the GBH-group). Results are presented as mean ± SEM. *p < 0.05.

**Figure 3:**
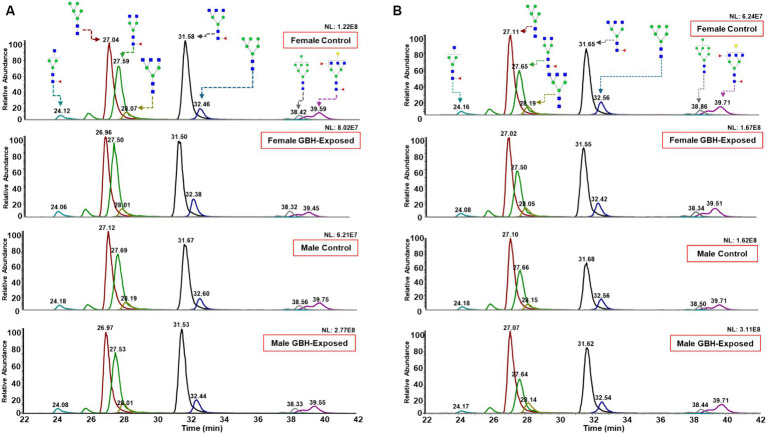
Representative Extracted Ion Chromatograms (EICs) of highly abundant *N*-glycans, comparing their expression in rat brain tissue between female GBH-exposed and female controls, male GBH-exposed and male controls in the **(A)** hippocampus and **(B)** prefrontal cortex. *N*-glycan nomenclature and composition as described in Figure 1. The different peak color codes represent the assigned glycan structure.

For this study, the *N*-glycan profile of the proteins present in the hippocampus and PFC of rat brain tissue exposed to GBH were investigated. The experimental workflow for the study approach is shown in Figure 1. Twelve SD rats, six males and six females, in the control group received daily oral doses of 1 mL of water per kg of body weight for 12 weeks. The GBH-treated group comprising 13 SD rats, six males and seven females, received oral doses of 100 mg of glyphosate/kg/day for the same duration. During the protocol neither food consumption nor body weight changed because of GBH-exposure as shown in Supplementary Figure S1. The effect of GBH on the episodic memory from the NOR test was investigated. As shown in Figure 2, the discrimination index of the different rat sex after 12 weeks of exposure to GBH or vehicle were recorded. The female rats chronically exposed to GBH showed a lower score in the discrimination index relative to the controls (*p* < 0.05), as shown in Figure 2A, while there was no significant difference between the GBH-exposed and control in the male category, as shown in Figure 2B. Subsequently, the rats were euthanized and their brain tissues were collected, then lysed to extract the proteins. An overnight PNGase F digestion was efficient for the release of the *N*-glycans (Huang and Orlando, 2017). A solid-phase permethylation reaction was conducted to enable separation of the *N*-glycans on the C18 column, enhance the stability of the *N*-glycan structures, and improve ionization efficiency. The online purification removed excess salts and impurities, which provided a better ionization of *N*-glycans. The LC–MS/MS and LC-ESI-MS-PRM methods offered high sensitivity, making the identification of the *N*-glycan structures possible. The identified peaks were manually confirmed and integrated using Skyline software. The identification of the *N*-glycan composition was achieved through full MS and MS^2^. Supplementary Figure S2 shows examples of *N*-glycan identification using the characteristic glycan oxonium ions with m/z values 468.2806, 783.9048, 656.3400, and 1029.0328. After the peak area integration, a normalization was carried out based on the total glycome abundance. The *N*-glycan profiles were compared amongst the studied cohorts; Figure 3A depicts the representative Extracted Ion Chromatograms (EICs) comparing the absolute intensity of the hippocampus section of the male and female brain in GBH-exposed and control groups; Figure 3B depicts the same comparison for the prefrontal region of the brain. A five-digit *N*-glycan nomenclature was used to simplify the structures, where “4-5-0-2-0” reads in the following order as *N*-acetylglucosamine, hexose (mannose or galactose), fucose, *N*-acetylneuraminic acid, and *N*-glycolylneuraminic acid (GlcNAc, Hex, Fuc, NeuAc, NeuGc). Glycan symbols are described in the caption of Figure 1.

### 3.2 Glycomics analysis of hippocampus section of the brain

**Figure 4:**
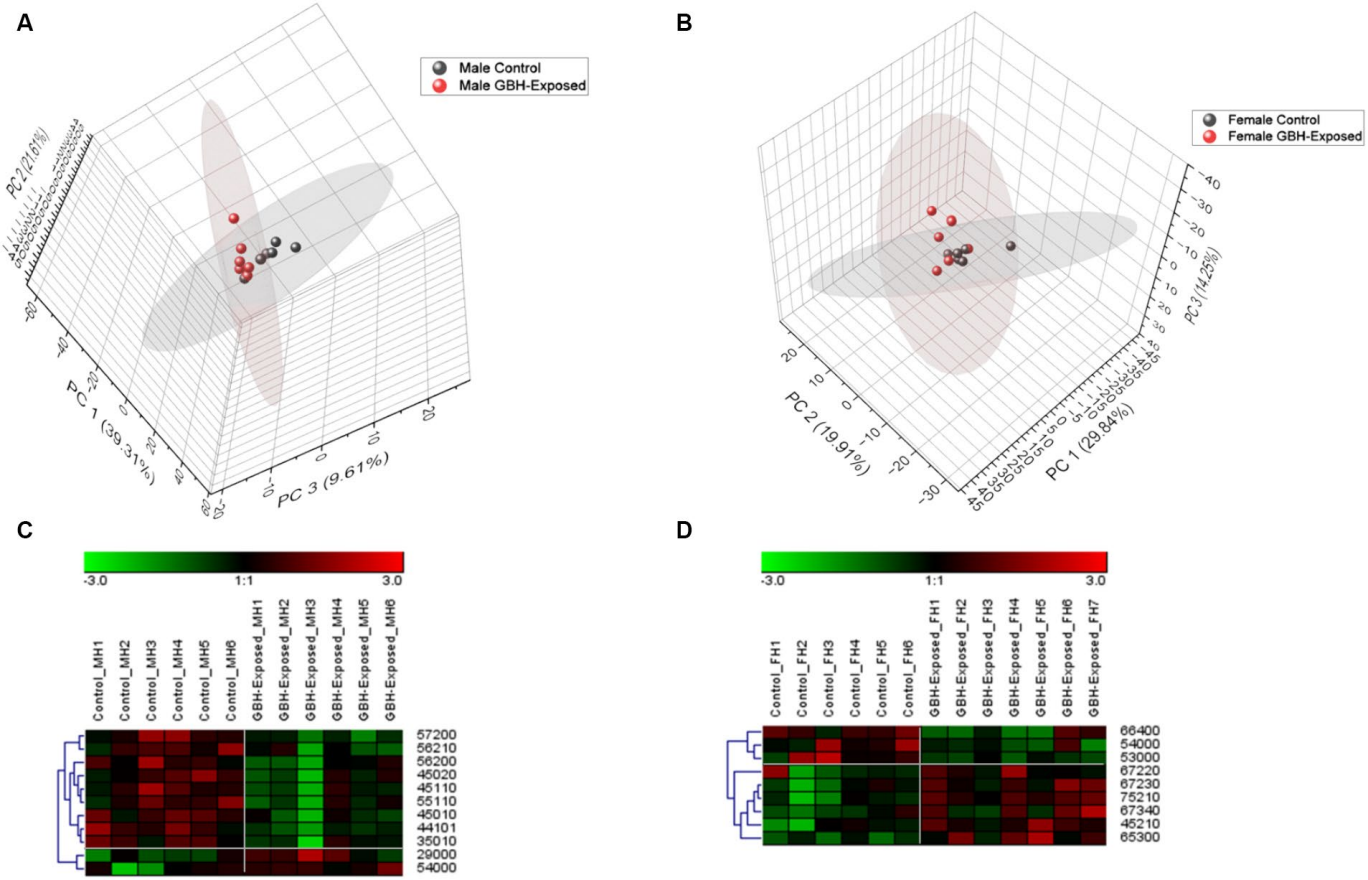
Unsupervised principal component analysis (PCA) with a confidence level of 95% showing all identified *N*-glycans and heatmap representation of statistically significant *N*-glycans in the hippocampus section of the brain. Comparing control vs GBH-exposed cohorts: **(A)** all identified *N*-glycans in the male cohort (*n* = 12); **(B)** all identified *N*-glycans in the female cohort (*n* = 13); **(C)** significant *N*-glycans in the male cohort; and **(D)** significant *N*-glycans in the female cohort.

A total of 125 *N*-glycans were identified and quantified in the hippocampus tissue, and principal component analysis (PCA) was utilized to compare and examine variations between the analyzed sample groups. PCA converts a complex set of observations with potentially correlated variables in high dimensions into a set of nonlinear values that are not correlated. This conversion was able to map our observations, and then show similarities among the different data sets (Abdi and Williams, 2010). Figures 4A,B show the 3D PCA plot generated from the identified glycans’ quantitative data. Unsupervised PCA was created using Origin Pro software, with a confidence level of 95%. Supplementary Figure S3 illustrates a PCA of combined gender data; Figure 4A displays the observed differences for males, whereas Figure 4B displays the observed differences for females. The differences in control and GBH-exposed groups for the males can be seen in the primary principal component 1 (PC1) and secondary principal component 3 (PC3), the same as for the females. This effect is significant for clustering when PC1 captures a substantial portion of the variance in the data that is relevant for distinguishing between clusters, thereby helping us track the glycome changes from a healthy state to the GBH-exposed state in rats. Heat maps were utilized to visually represent the significant differences between the two cohorts, as seen in Figures 4C,D. The heat map depicts the statistically significant *N*-glycans between control relative to GBH-exposed cohort in the male and female gender subgroup for the hippocampus. The study also examined variations in the expression of *N*-glycans in each cohort using the Mann–Whitney U test and Benjamini-Hochberg correction. Their *p* values, adjusted *p* values, fold change (FC), and expression level are noted in Supplementary Table S2 for the male cohorts and Supplementary Table S3 for the female cohorts. The results indicated that eleven *N*-glycans showed statistically significant differences in the comparison between the control and GBH-exposed rats in the male cohorts; nine were downregulated and two were upregulated in the GBH-exposed group. In the female cohorts, nine *N*-glycans were statistically significant; three were downregulated while six were upregulated. The expressions of the different *N*-glycan types, including high mannose, fucosylated, sialofucosylated, sialylated NeuAc, sialylated NeuGc, and other entities, were identified and compared across the control and GBH-exposed cohorts as shown in Supplementary Figure S4. The fucosylated glycans were the most prevalent. There was an observed increase in the relative abundance of the fucosylated *N*-glycans in the GBH-exposed cohorts as compared to the controls, across all comparisons. An increase was observed in the female cohorts and a decrease in the male cohorts for sialofucosylated glycans. Similar patterns of increase and decrease were observed in the high mannose for both comparisons. There was an observed significant difference in the sialylated NeuAc glycan type in the male cohorts.

**Figure 5:**
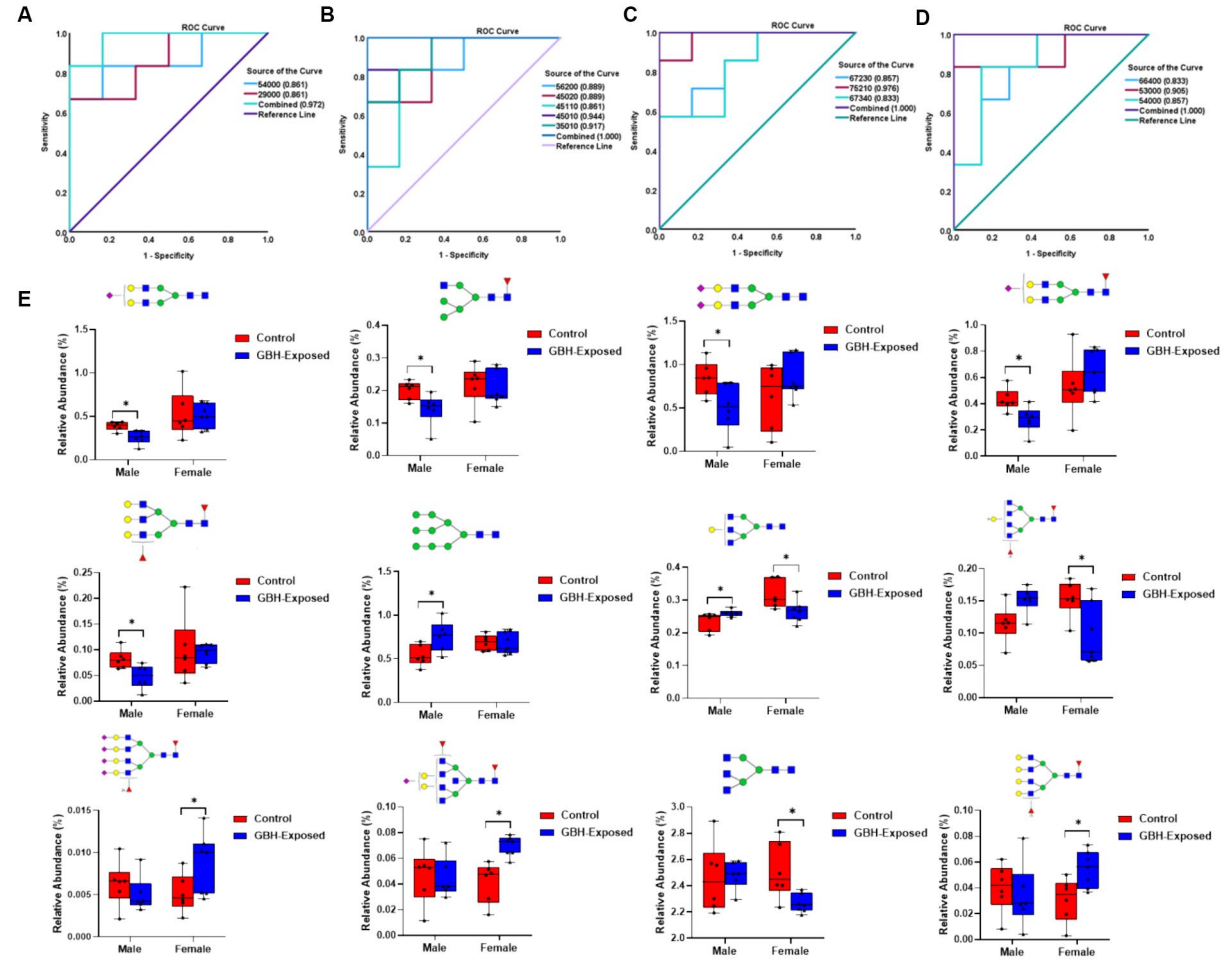
ROC/AUC curves of PRM-validated statistically significant *N*-glycans in the hippocampus brain tissue **(A)** male group ROC curve for upregulated *N*-glycans, and **(B)** male group ROC curve for downregulated *N*-glycans, and **(C)** female group ROC curve for upregulated *N*-glycans, and **(D)** female group ROC curve for downregulated *N*-glycans. **(E)** Box graphs of the relative abundance of the PRM-validated glycans that displayed significant differences present in the gender subgroups of the hippocampus section of the brain after comparing the GBH-exposed to control (* represents *p* value <0.05, ** represents *p* value <0.01). *N*-glycan nomenclature and composition as described in Figure 1.

To assess the reliability of the identified *N*-glycan expressions as potential biomarkers, a receiver operating characteristic (ROC) curve analysis was employed, as depicted in Figure 5. ROC curves are commonly used to determine the accuracy of tests by calculating the areas under the curve (AUCs) (Zweig and Campbell, 1993). AUC values closer to 0.5 imply poor diagnostic performance, whereas values closer to 1.0 reflect strong diagnostic accuracy (Obuchowski and Bullen, 2018). The investigation for determining the AUC values for each of the PRM-validated structures is depicted in Figures 5A–D. For the upregulated *N*-glycans in the male group, GlcNAc_5_Hex_4_ and GlcNAc_2_Hex_9_ displayed an AUC of 0.86 with a combined AUC of 0.97 as shown in Figure 5A. For the downregulated *N*-glycans in the male group, GlcNAc_5_Hex_6_Fuc_2_ and GlcNAc_4_Hex_5_NeuAc_2_ displayed an AUC of 0.89, GlcNAc_4_Hex_5_Fuc_1_NeuAc_1_ displayed an AUC of 0.86, GlcNAc_4_Hex_5_NeuAc_1_ displayed an AUC of 0.94, and GlcNAc_3_Hex_5_NeuAc_1_ displayed an AUC of 0.92, with a combined AUC of 1.00 as shown in Figure 5B. For the upregulated glycans in the female group, GlcNAc_6_Hex_7_Fuc_2_NeuAc_3_ displayed an AUC of 0.86, GlcNAc_7_Hex_5_Fuc_2_NeuAc_1_ displayed an AUC of 0.98, and GlcNAc_6_Hex_7_Fuc_3_NeuAc_4_ displayed an AUC of 0.83, with a combined AUC of 1.00 as shown in Figure 5C. For the downregulated glycans in the female group, GlcNAc_5_Hex_3_ displayed an AUC of 0.91, GlcNAc_5_Hex_4_ displayed an AUC of 0.86, and GlcNAc_6_Hex_6_Fuc_4_ displayed an AUC of 0.83, with a combined AUC of 1.00 as shown in Figure 5D. The combined AUCs show that the female cohorts are more distinguishable as compared to the male. The box plots shown in Figure 5E represent the PRM-validated statistically significant glycans present in the gender subgroups of the hippocampus section of the brain. GlcNAc_5_Hex_4_ was statistically significant in both genders, being upregulated in the male GBH-exposed rats and downregulated in the female GBH-exposed rats. This indicates its expression in the male was different from its expression in female. In total, seven *N*-glycans were PRM-validated for the male cohorts; five were under-expressed, and two were overexpressed. For the female cohorts, six *N*-glycans were PRM-validated; three were over-expressed while three were under-expressed.

### 3.3 Glycomics analysis of prefrontal cortex section of the brain

**Figure 6:**
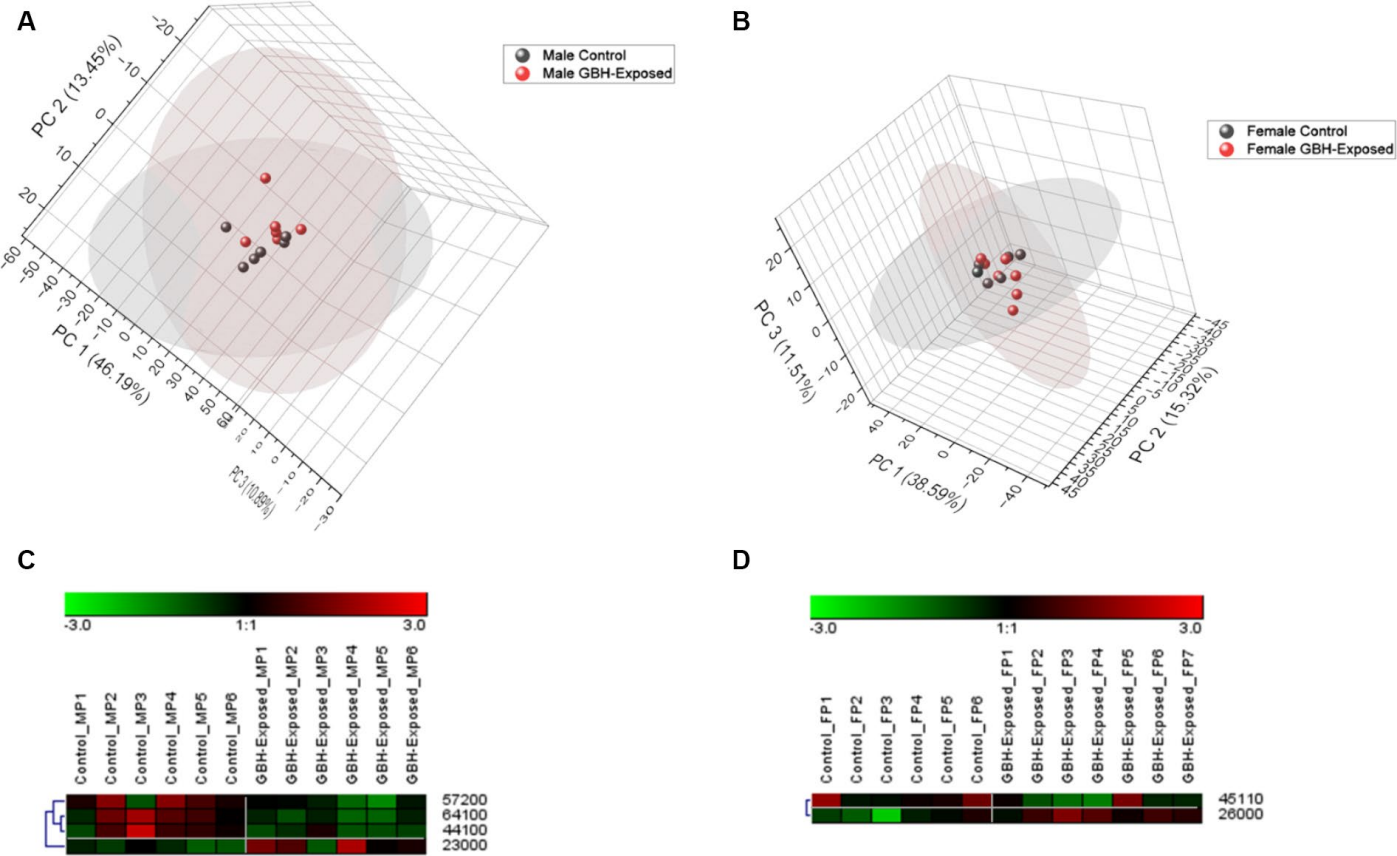
Principal component analysis (PCA) with a confidence level of 95% of all identified *N*-glycans and heatmap representation of statistically significant *N*-glycans in the prefrontal cortex section of the brain. Comparing control vs. GBH-exposed cohorts: **(A)** all identified *N*-glycans in the male cohorts (*n* = 12); **(B)** all identified *N*-glycans in the female cohorts (*n* = 13); **(C)** significant *N*-glycans in the male cohorts; and **(D)** significant *N*-glycans in the female cohorts.

A total of 125 *N*-glycans were identified and quantified in the PFC tissue sample; Supplementary Figure S5 illustrates a PCA of combined gender data. Figure 6A displays the observed differences for males, whereas Figure 6B displays the observed differences for females. The disparities between the control and GBH-exposed groups among males are evident in the primary principal component 1 (PC1) and secondary principal component 2 (PC2), just as they are for females. This enables us to monitor the alterations in the *N*-glycome from a normal condition to the state of exposure to GBH in rats. Heat maps were utilized to visually represent the differences between the two cohorts, as seen in Figures 6C,D. Statistically significant *N*-glycans between control relative to GBH-exposed cohort in the PFC with their *p* values, adjusted *p* value, fold change (FC), and expression level are noted in Supplementary Table S4 for the male cohorts and Supplementary Table S5 for the female cohorts. The results showed four *N*-glycans with statistically significant differences in expression in the comparison between the control and GBH-exposed male cohorts: three were downregulated and one was upregulated in the GBH-exposed group. For the comparison in the female cohorts, two *N*-glycans were statistically significant: one was downregulated while one was upregulated. The expressions of the different *N*-glycan types were identified and compared across the control and exposed as shown in Supplementary Figure S6. The fucosylated glycans were the most prevalent. There was an observed decrease in the relative abundance of the fucosylated glycans in the GBH-exposed cohorts as compared to the controls, across all comparisons. There was an increase in the relative abundance of sialylated NeuGc *N*-glycans for both genders, and similar results were also observed in the high mannose *N*-glycan type.

**Figure 7:**
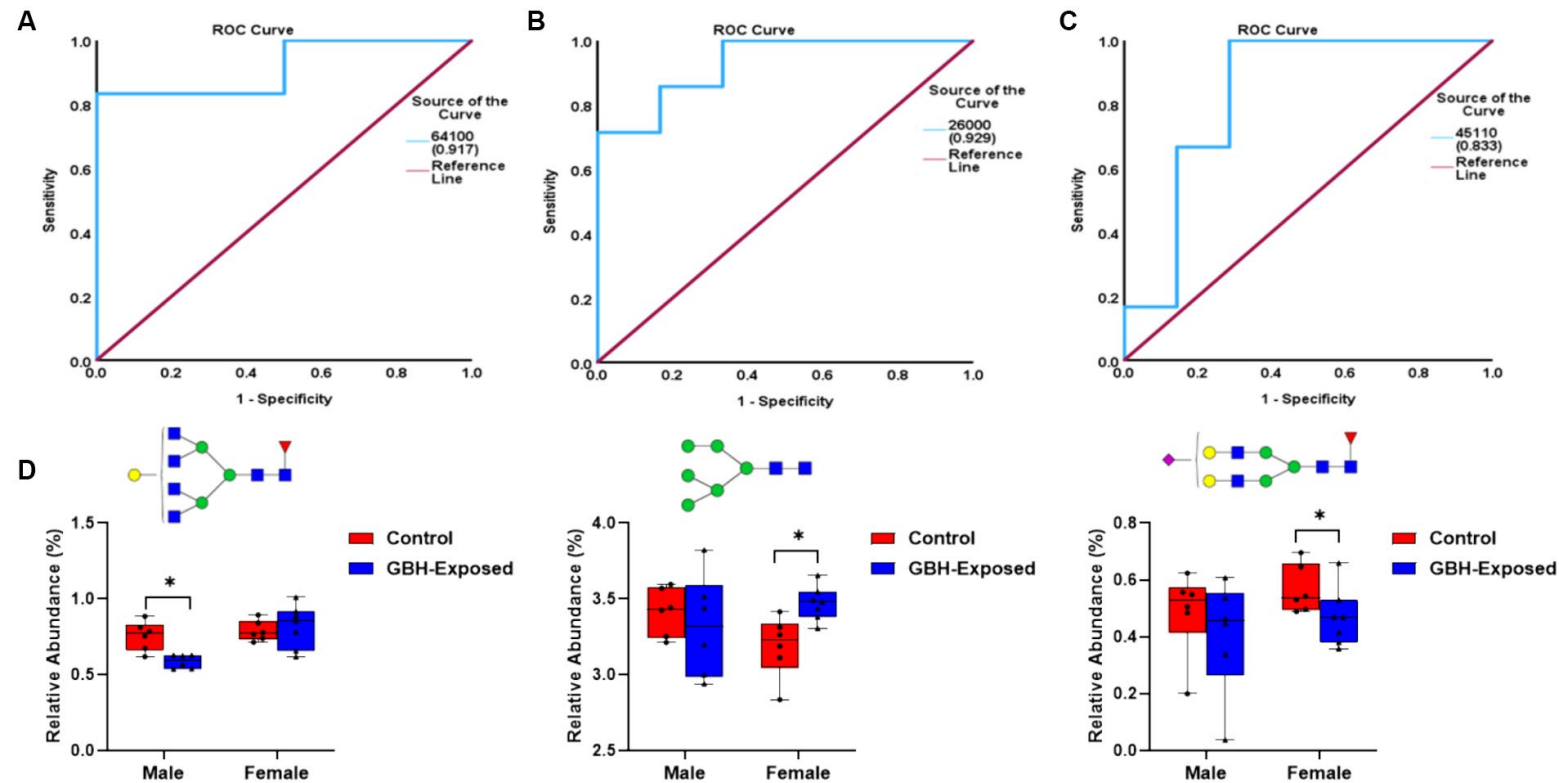
ROC/AUC curves of the PRM-validated statistically significant *N*-glycans in the prefrontal cortex brain tissue: **(A)** male group ROC curve for downregulated *N*-glycans, **(B)** female group ROC curve for upregulated *N*-glycans, and **(C)** female group ROC curve for downregulated *N*-glycans. **(D)** Box graphs representing the PRM-validated statistically significant glycans present in the gender subgroups of the prefrontal cortex section of the brain after comparing the GBH-exposed to control (* represents *p* value <0.05 and ** represents *p* value <0.01).

The ROC/AUC curves generated with the PRM-validated significant *N*-glycans in the PFC are shown in Figure 7. The process involved determining the AUC values for each PRM-validated *N*-glycan, as depicted in Figures 7A–C. For the downregulated *N*-glycan in the male group, GlcNAc_6_Hex_4_Fuc_1_ displayed an AUC of 0.92 as shown in Figure 7A. For the upregulated *N*-glycan in the female group, GlcNAc_2_Hex_6_ displayed an AUC of 0.93 as shown in Figure 7B. For the downregulated *N*-glycan in the female group, GlcNAc_4_Hex_5_Fuc_1_NeuAc_1_ displayed an AUC of 0.83, as shown in Figure 7C. Comparing the AUCs shows that the female cohort is more distinguishable as compared to the male. The box plots of the aforementioned *N*-glycans are depicted in Figure 7D. The *N*-glycan GlcNAc_6_Hex_4_Fuc_1_ was PRM-validated for the male cohorts, and it was under-expressed. For the female cohorts, the *N*-glycans GlcNAc_2_Hex_6_ and GlcNAc_4_Hex_5_NeuAc_1_ were PRM-validated. The GlcNAc_2_Hex_6_ was over-expressed while the GlcNAc_4_Hex_5_NeuAc_1_ was under-expressed.

## 4 Discussion

Substantial evidence has suggested the neurotoxic effects of glyphosate and GBH on memory and behavior as the main cognitive functions impaired after chronic or subchronic exposures to these substances (Gallegos et al., 2018; Ait-Bali et al., 2020; Bicca et al., 2021). In addition, several molecular mechanisms possibly associated with alterations in cognition and behavior due to intoxication with glyphosate have been reviewed recently (Chávez-Reyes et al., 2024a). In this sense, the role of glyphosate on neurodevelopment has been poorly explored. Alteration in anxiety levels and memory in rats exposed orally to GBH during the developmental stage showed depressive-like behavior (Cattani et al., 2017). In addition, offsprings from Wistar rat exposed daily to GBH throughout the entire gestation period resulted in alterations in behavior and memory (de Oliveira et al., 2022). These alterations could be explained, at least in part, by; alteration in neurotransmission, since perinatal exposition to GBH in rat offspring produced an increment in glutamate release (Cattani et al., 2014, 2017); oxidative stress, since the perinatal exposure to GBH induced oxidative stress in the hippocampus and PFC from offspring Wistar rats (de Oliveira et al., 2022); or neuroinflammation, since mice offspring exposed to GBH during gestation showed an increase in TNF-α in the PFC (De Castro Vieira Carneiro et al., 2023). Exposition to GBH in early stages of the development may therefore drive alterations via modification in key signaling pathways, affecting neuronal growth and migration in the central and peripheral nervous systems (Costas-Ferreira et al., 2022).

With limited evidence regarding the neurotoxic effects of GBH at the posttranscriptional level, this study was designed to assess the impact of chronic GBH intoxication on glycome profiles in two key brain structures associated with memory and behavior: the PFC and hippocampus (Preston and Eichenbaum, 2013). The neurotoxic effects of GBHs were assessed by analyzing the brain *N*-glycome of rats chronically exposed to GBHs, providing a more comprehensive understanding of their impact. This study presents, for the first time, a detailed analysis of the *N*-glycome profiles in two brain regions associated with cognition, the hippocampus and the PFC of rats that were exposed to GBH for 12 weeks. The results of our study reveal alterations in the *N*-glycome profile and their role in the neurotoxic effects induced by exposure to GBH. Research has shown that herbicide exposure could be a risk factor for neurotoxicity and neurodegenerative disorders (Manning-Bog et al., 2002; McCormack et al., 2002; Chen et al., 2012; Cattani et al., 2014). Hence, we evaluated the effect of GBH on both the hippocampus and PFC of the brain. According to our findings, discussed further below, GBH had a greater impact on the hippocampus compared to the PFC. The hippocampus appeared to be more vulnerable to GBH toxicity. The neural pathways connecting both brain regions have been shown to facilitate bidirectional communication during memory processing in mice (Rajasethupathy et al., 2015). The glycome profiles in both brain regions were found to be distinct, as evidenced by the data presented in Supplementary Tables S2–S5. This divergence likely mirrors the dynamic and intricate nature of the brain’s glycome. Elucidating these distinctions is crucial for deciphering the mechanisms that govern brain function and dysfunction, thereby offering valuable insights into the regional nuances of brain function, development, and disease pathogenesis.

A sex-specific effect has been observed on exposure to toxins (Giommi et al., 2022). Sex differences have been shown to influence many parts of the brain and behavior including memory, emotion, pain perception, neurotransmitter levels, and otoacoustic emissions (McFadden, 2000). They are present across all brain lobes, including various cognitive regions such as the hippocampus and amygdala (Gorski et al., 1978, 1980; Juraska, 1991; Goldstein et al., 2001). The ratios of grey matter to white matter exhibit considerable variations between males and females in several regions of the human cortex (Allen et al., 2003). One region that clearly exhibits sexual dimorphism in both its anatomy and function is the hippocampus (Madeira and Lieberman, 1995; Goldstein et al., 2001). Within the hippocampus, sex differences are also found in many neurotransmitter systems (Teyler et al., 1980; Turner, 1985; Romeo et al., 2005). Sex differences were also studied in the PFC, with major changes observed (Speck et al., 2000). The events that cause sex variations in disease progression are still yet to be well understood (Hanamsagar and Bilbo, 2016). Importantly, female rats chronically exposed to GBH in the present study showed poor exploration of the novel object in the NOR test (Figure 2). As rodents have a strong innate inclination for exploring novel objects over the already known ones (Antunes and Biala, 2012; Lueptow, 2017), this low discrimination index detected in female rats may suggest a deep impairment into the episodic memory mechanisms and/or brain related areas (especially, the hippocampus) (Broadbent et al., 2010; Dickerson and Eichenbaum, 2010). This could also suggest a difference in cognitive function, perception, or response to stimuli, whereas male rats remain unaltered.

GBH increased some *N*-glycan expression in both regions of the brain in both males and females. Most of these upregulated effect(s) were more specific to the females. In this study, we examined the *N*-glycome in homogenates from the hippocampus and PFC of the brain of GBH-exposed and control rats using LC–MS/MS of permethylated *N*-glycans, to identify specific glyco-signatures. A total of 125 *N*-glycans were identified in both compartments of the rat brain tissue, encompassing all glycan types, including high mannose, fucosylated, sialofucosylated, sialylated NeuAc, sialylated NeuGc, and other entities. An elevation in fucosylated glycans the hippocampus region was detected in the cohorts exposed to GBH, in comparison to the control group, as depicted in Supplementary Figure S4. Conversely, a decrease in fucosylated glycans was observed in the PFC, as illustrated in Supplementary Figure S6. In the literature, complex structures such as fucosylated, sialofucosylated, and oligomannose structures such as high mannose have shown to be of high abundance in the brain (Shimizu et al., 1993; Gizaw et al., 2016; Gaunitz et al., 2021), which is in line with our data. They have been studied for their role in disease progression, and their aberrant expression has also been implicated in neurodegenerative conditions (Gizaw et al., 2016; Gaunitz et al., 2021). The inherent variety of glycan structures allows glycans to convey specific information that is recognized by receptors and translated into a particular biological function. Core fucosylated glycans have been proven to be altered in disease conditions (Miyoshi et al., 1999; Noda et al., 2003). Further to this study, additional research is needed to investigate the reasons for changes and the impact of glycosylation in various regions of the brain affected by GBH. This is crucial for comprehending the connection between protein glycosylation and neuropathology from GBH toxicity.

PCA helps to track changes in the total *N*-glycome observed in a healthy rat relative to a GBH-exposed rat. Supplementary Figure S3 shows the PCA of combined gender data, while Figures 4A,B show the differences observed in the males and females, respectively, for the hippocampus. The controls and GBH-exposed cohorts’ clusters were indistinguishable when the genders were merged to create a unified PCA plot. Astonishingly, when the PCA was plotted using data from both genders, it was unable to differentiate between the control group and the group that was exposed to GBH. The gender disparities between the control and GBH-exposed groups were evident in the PCA plots, which were generated separately based on gender. The same was observed in the PFC. Supplementary Figure S5 shows a PCA of combined gender data observed in the PFC. Figure 6A shows the observed differences for males, while Figure 6B shows the differences observed for females. The PCA demonstrated distinct variations in the response of GBHs based on sex, indicating the animals that were most vulnerable to the harmful impacts of GBH. Therefore, the findings derived from the PCA validate that when both genders were combined, the control and GBH-exposed cohorts were indistinguishable. However, when the genders were plotted individually, distinct disparities became apparent. This phenomenon is likely a result of sexual dimorphism, which can be related to the differential responses of genders to GBH.

The Venn diagram illustrated in Supplementary Figure S7 provides a visual depiction of the common and distinct significant *N*-glycans among the different cohorts and regions, employing intersecting circles for comparison. This facilitates comprehension of the glycan profiles within each subgroup and underscores potential similarities and disparities in glycan expression patterns between males and females for each brain region. On comparing the male and the female, a common glycan was seen in both genders in the hippocampus compartment of the brain as shown in Supplementary Figure S7A. GlcNAc_5_Hex_4_ was significant across both genders. It was overexpressed in the male and underexpressed in the female, as shown in Figures 4C,D. Between the male and the female cohorts in the same brain compartment, ten *N*-glycans were unique to the males and eight *N*-glycans unique to the females, as shown in Supplementary Figure S7A. In the PFC, no *N*-glycan overlap appeared; however, the male cohorts exhibited four distinct *N*-glycans, while the female cohorts displayed two distinct glycans, as depicted in Supplementary Figure S7B. Additionally, a comparison of the N-glycans in the two compartments was conducted. Upon comparison of both regions in the male cohorts, one significant *N*-glycan was common to both the hippocampus and the PFC: GlcNAc_5_Hex_7_Fuc_2_. The observed pattern of underexpression was consistent throughout both regions. This *N*-glycan is also known as a bisecting *N*-glycan. According to reports, these have been implicated in neurological disorders. Neurological difficulties arise from a deficit in *N*-acetylglucosaminyltransferase III (GnT-III), whereas higher production promotes neurite formation by incorporating bisecting GlcNAc into *N*-glycans (Gaunitz et al., 2021). Of the three significant *N*-glycans unique to the PFC region of the male cohort brain as indicated in Supplementary Figure S7C, two glycans, GlcNAc_6_Hex_4_Fuc_1_ and GlcNAc_4_Hex_4_Fuc_1_, were found to be core fucosylated. The position of the fucose was confirmed through MS2 as shown in Supplementary Figure S2. They were underexpressed in this region of the brain. Core fucosylated *N*-glycan structures have shown potential as biomarkers for cancer diagnosis, prognosis, and treatment monitoring (Liao et al., 2021; Tan et al., 2023). FUT8 is a member of the fucosyltransferase family and plays a crucial role as the primary enzyme in *N*-glycan core fucosylation (Miyoshi et al., 2008). Therefore, targeting critical core fucosylated glycans could be a valuable diagnostic tool for neurotoxicity.

The significant high mannose glycans present in the male cohort (GlcNAc_2_Hex_3_) as shown in Figure 6C and female (GlcNAc_2_Hex_6_) cohort of the PFC as shown in Figure 6D were overly expressed. High mannose glycans have been reported to play a role in brain development and can be found in neural synapses (Gaunitz et al., 2021). Their elevated level has also been reported to play a role in breast cancer progression (Ščupáková et al., 2021). The heightened abundance of high mannose glycans impacts the protein’s functionality. It can change the protein’s stability, its interaction with substrates, its half-life in the blood, and its adhesion and communication properties (De Leoz et al., 2011). This could potentially be examined as a characteristic feature for neurotoxicity following exposure to GBH. The observed upregulation and downregulation of *N*-glycan levels suggests that *N*-glycosylation could be a promising area of investigation for GBH exposure. The Venn plot in Supplementary Figure S6C reveals that there are ten distinct glycans that are specifically found in the hippocampal region of the male brain cohort. Of these ten, the observed phenomenon of core fucosylation in the glycan GlcNAc_5_Hex_6_Fuc_2_ is consistent with the observed pattern of downregulation in fucosylated glycans (distinct) in the PFC region of the male cohorts. Three sialofucosylated glycans, GlcNAc_4_Hex_5_Fuc_1_NeuAc_1_, GlcNAc_5_Hex_5_Fuc_1_NeuAc_1_, and GlcNAc_5_Hex_6_Fuc_2_NeuAc were downregulated. Sialofucosylated glycas have been implicated as significantly altered in neurodegenerative diseases, such as Alhzhemier’s Disease, which supports their potential for use as relevant biomarkers (Reyes et al., 2022). Therefore, it will be interesting to investigate in future studies how these *N*-glycans are mechanistically related to neurotoxicity. Three significant *N*-glycans were unique to the prefrontal region of the male cohort brain as indicated by the Venn plot in Supplementary Figure S7C: two of these are fucosylated *N*-glycans, underexpressed in the GBH-exposed as compared to the control; the third was a high mannose *N*-glycan. The details are shown in Supplementary Tables S6–S10. The presence of sialofucosylated *N*-glycans was predominantly noted in both regions of the female cohorts, as indicated in Supplementary Figure S7D and depicted in Figures 4D, 6D. This observation may facilitate the development of diagnostic tools for neurotoxicity.

A targeted study, known as PRM validation, was conducted on the significant *N*-glycans that were identified in the untargeted analysis. Targeted mass spectrometry is a powerful method for performing a quantitative study of biomolecules (Cho et al., 2022). The high sensitivity and specificity of the approach have contributed to its widespread use in omics research. It allows for simultaneous detection of MS/MS transitions in a single experiment by utilizing Orbitrap instruments, which enhances the quantitative analysis of biomarkers for diverse diseases. Due to the significance of glycans as possible biomarkers, there is a need for enhanced and focused methods to efficiently measure the glycan quantities (Lin et al., 2022). After validation in this study, ROC curves were utilized to offer insights into the specificity and accuracy of the subject as a potential diagnostic sign for the disease state, thereby examining the reliability of these glycan expressions as possible biomarkers. The ROC plot is presented in Figures 5A,B (male), and Figures 5C,D (female) for the hippocampus brain tissue. Figure 7A (male) and Figures 7B,C (female) show the ROC plots for the PFC. The results indicate that when genders are examined individually, the *N*-glycans have the ability to accurately differentiate between the control and GBH-exposed groups in both brain areas. This result further supports the significance of these glycans in the process of uncovering possible biomarkers. Box graphs were plotted for the significant PRM *N*-glycans to aid the visualization of the over- and underexpression of individual signals, as shown in Figure 5E for the hippocampus and Figure 7D for the PFC.

## 5 Conclusion

In this work, we performed comprehensive *N*-glycan profiling from the brain tissue of rats exposed to GBHs. This was accomplished using several techniques which involved release of the *N*-glycans, purification using C18 cartridges, reduction, permethylation, nanoflow reverse-phase liquid chromatography, and highly sensitive mass spectrometry. This process was applied to all samples. The *N*-glycan profiles of the hippocampus of the GBH-exposed rats were compared to the controls. The same was compared for the PFC. Sex differences were studied in both regions of the brain, where major changes were observed in *N*-glycan expression. Using PCA, *N*-glycan profiles of the GBH-exposed rats were distinguishable from the controls when the samples were analyzed separately by gender, as compared to the combined gender PCA. ROC-AUC analysis was used to assess the reliability of the significant *N*-glycans, and to determine the AUC values for each PRM-validated *N*-glycan structure. The data shows the cohorts are distinguishable. This therefore suggests that gender plays a role in disease conditions and, in this case, in the neurotoxic effect of GBH. Interestingly, episodic memory was impaired in female rats exposed chronically to GBH, whereas the episodic memory of the male rats remained unaltered. This may suggest a higher susceptibility in the female brain (possibly at the hippocampal level), which could be related to the gender glycomic profile observed. Moreover, a high abundance of fucosylated, sialofucosylated, and high mannose structures were observed in both regions of the brain. These glycome changes are associated with many biological functions. Some of the significant *N*-glycans have been previously found to be associated with neurological impairment and cancer, among other pathologies. Therefore, the identified alterations in these *N*-glycan types indicate a possible neurotoxic impact associated with chronic exposure to GBH. The recognition of these modified *N*-glycans provides significant markers that may enhance comprehension of the mechanisms involved in GBH-induced toxicity and its potential ramifications for neurological well-being. *N*-glycans could therefore be a potential diagnostic tool for disease studies. Understanding the mechanisms underlying sex differences may aid in the development of more personalized therapies with a better success rate, particularly in diseases where sex differences are most prevalent.

## Data availability statement

The datasets presented in this study can be found in online repositories. The names of the repository/repositories and accession number(s) can be found at: https://glycopost.glycosmos.org/, GPST000408.

## Ethics statement

The animal study was approved by the experimental procedures adhered to the Mexican Guidelines for Animal Care NOM-062-ZOO-1999 and the National Research Council Guide for the Care and Use of Laboratory Animals. The study was conducted in accordance with the local legislation and institutional requirements.

## Author contributions

JS: Data curation, Formal analysis, Investigation, Methodology, Writing – original draft. CG-R: Data curation, Formal analysis, Investigation, Methodology, Writing – original draft. JC-R: Data curation, Investigation, Methodology, Writing – review & editing. SO: Data curation, Formal analysis, Writing – review & editing. BM-C: Data curation, Formal analysis, Investigation, Writing – review & editing. CL-L: Data curation, Formal analysis, Methodology, Writing – review & editing. MB: Data curation, Writing – review & editing. YM: Conceptualization, Funding acquisition, Project administration, Resources, Supervision, Writing – review & editing.
