# Supplementary material for: Neuroglycome alterations of hippocampus and prefrontal cortex of juvenile rats chronically exposed to glyphosate-based herbicide
Source: Front Neurosci. 2024 Aug 21;18:1442772. doi: 10.3389/fnins.2024.1442772 (PMC11371619; doi:10.3389/fnins.2024.1442772)
Supplement: Supplementary file 1 [file Data_Sheet_1.docx]

Supplementary Material

# Supplementary Data Table of Contents

**Supplementary Figures**

**Figure S-1:** Body weight changes during GBH exposure in (a) Females and (b) Males.

**Figure S-2:** Representative *N-*glycan structure showing the Extracted Ion Chromatogram of the *N*-glycan composition (a) GlcNAc_6_Hex_4_Fuc_1_ (b) GlcNAc_4_Hex_4_Fuc_1._ Inset is the full MS and the Mass Spectra 2 (MS/MS) of the structure. The *N-*glycan composition:
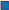
 HexNAc, 
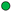
 
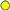
 Hex, 
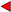
 Fuc, 
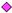
 NeuAc,
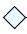
 NeuGc.

**Figure S-3:** Principal component analysis (PCA) with a confidence level of 95% of all identified *N*-glycans in the hippocampus section of the brain. Comparing Control vs GBH-Exposed cohorts; combined gender data (N=25).

**Figure S-4:** Distribution of all identified *N*-glycans by types between Control vs GBH-Exposed cohorts in (a) Male and (b) Female gender subgroups of the hippocampus section of the brain.

**Figure S-5:** Principal component analysis (PCA) with a confidence level of 95% of all identified *N*-glycans in the prefrontal cortex section of the brain. Comparing Control vs GBH-Exposed cohorts; combined gender data (N=25).

**Figure S-6:** Distribution of all identified *N*-glycans by types between Control vs GBH-Exposed cohorts in (a) Male and (b) Female gender subgroups of the prefrontal cortex section of the brain.

**Figure S-7:** Venn plot showing unique and overlapping significant *N*-glycans: in the gender subgroup data sets in (a) hippocampus section of the brain (b) prefrontal cortex section of the brain, and in the gender subgroup data sets between the hippocampus *vs* prefrontal cortex (c) Male (d) Female.

**Supplementary Tables:**

**Table S-1:** *N-*glycans in the male gender rat subgroup of the hippocampus section of the brain validated by LC-PRM-MS, including precursor m/z, transition fragment ions, fold change (FC), and log2fc for the full scan and PRM validation.

**Table S-2:** Statistically significant N-glycans between control relative to GBH-Exposed cohort in the male gender subgroup for hippocampus with their p-values, adjusted p-value, fold change (FC), and expression level. *N-*glycan nomenclature and composition as described in **Figure S-2**.

**Table S-3:** Statistically significant N-glycans between control relative to GBH-Exposed cohort in the female gender subgroup for hippocampus with their p-values, adjusted p-value, fold change (FC), and expression level. *N-*glycan nomenclature and composition as described in **Figure S-2**.

**Table S-4:** Statistically significant N-glycans between control relative to GBH-Exposed cohort in the male gender subgroup for prefrontal cortex with their p-values, adjusted p-value, fold change (FC), and expression level. *N-*glycan nomenclature and composition as described in **Figure S-2**.

**Table S-5:** Statistically significant N-glycans between control relative to GBH-Exposed cohort in the female gender subgroup for prefrontal cortex with their p-values, adjusted p-value, fold change (FC), and expression level. *N-*glycan nomenclature and composition as described in **Figure S-2**.

**Table S-6:** Significant *N-*glycans common and unique between the gender cohorts of the hippocampus section of the brain.

**Table S-7:** Significant *N-*glycans common and unique between the gender cohorts of the prefrontal cortex section of the brain.

**Table S-8:** Significant *N-*glycans common and unique in the combined group comparing the hippocampus section to the prefrontal cortex of the brain.

**Table S-9:** Significant *N-*glycans common and unique in the male gender rat group comparing the hippocampus section to the prefrontal cortex of the brain.

**Table S-10:** Significant *N-*glycans common and unique in the female gender rat group comparing the hippocampus section to the prefrontal cortex of the brain.

## Supplementary Figures

**Figure S-1:** Body weight changes during GBH exposure in (a) Females and (b) Males.


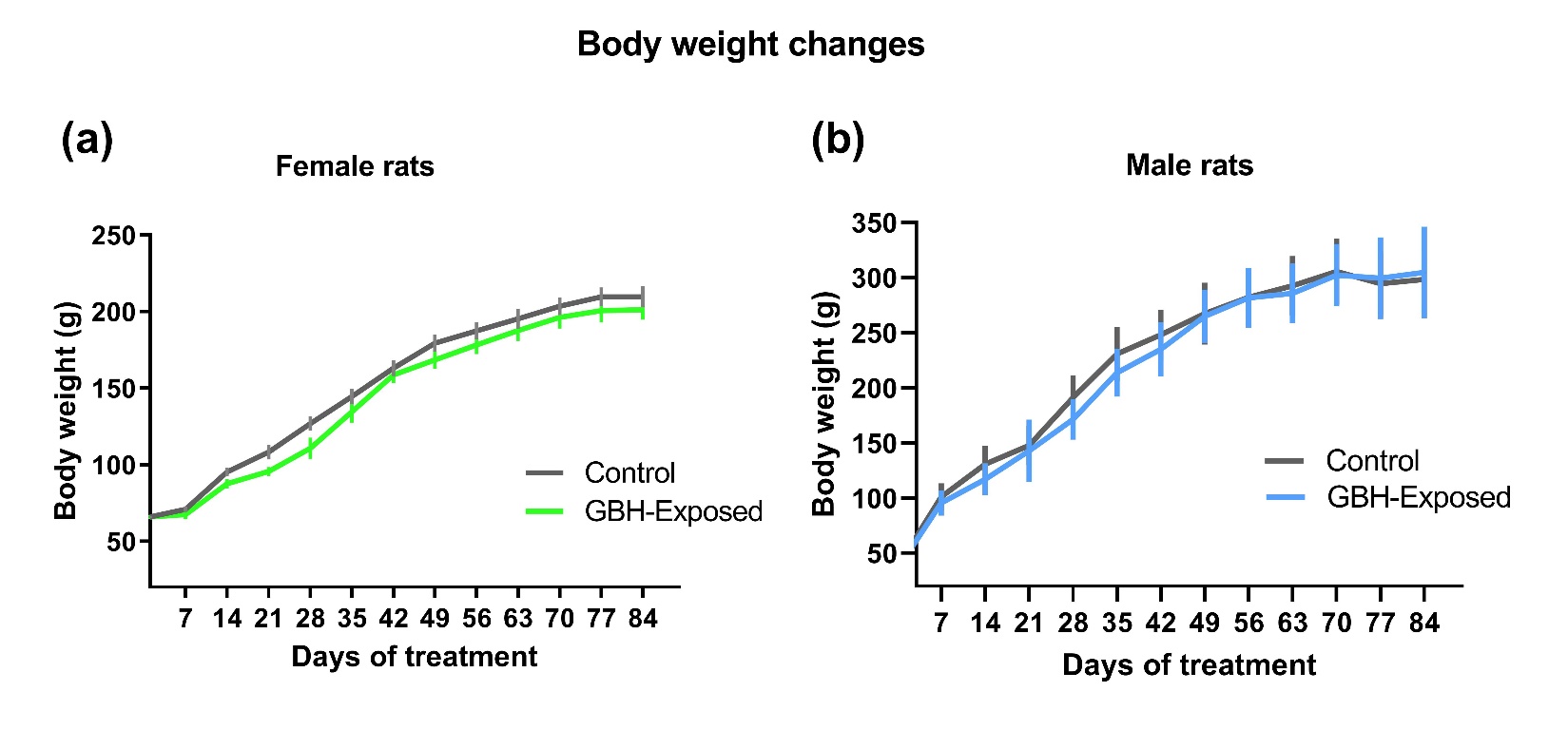


**Figure S-2:** Representative *N-*glycan structure showing the Extracted Ion Chromatogram of the *N*-glycan composition (a) GlcNAc_6_Hex_4_Fuc_1_ (b) GlcNAc_4_Hex_4_Fuc_1._ Inset is the full MS and the Mass Spectra 2 (MS/MS) of the structure. The *N-*glycan composition:
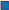
 HexNAc, 
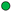
 
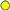
 Hex, 
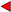
 Fuc, 
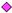
 NeuAc,
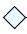
 NeuGc.

**
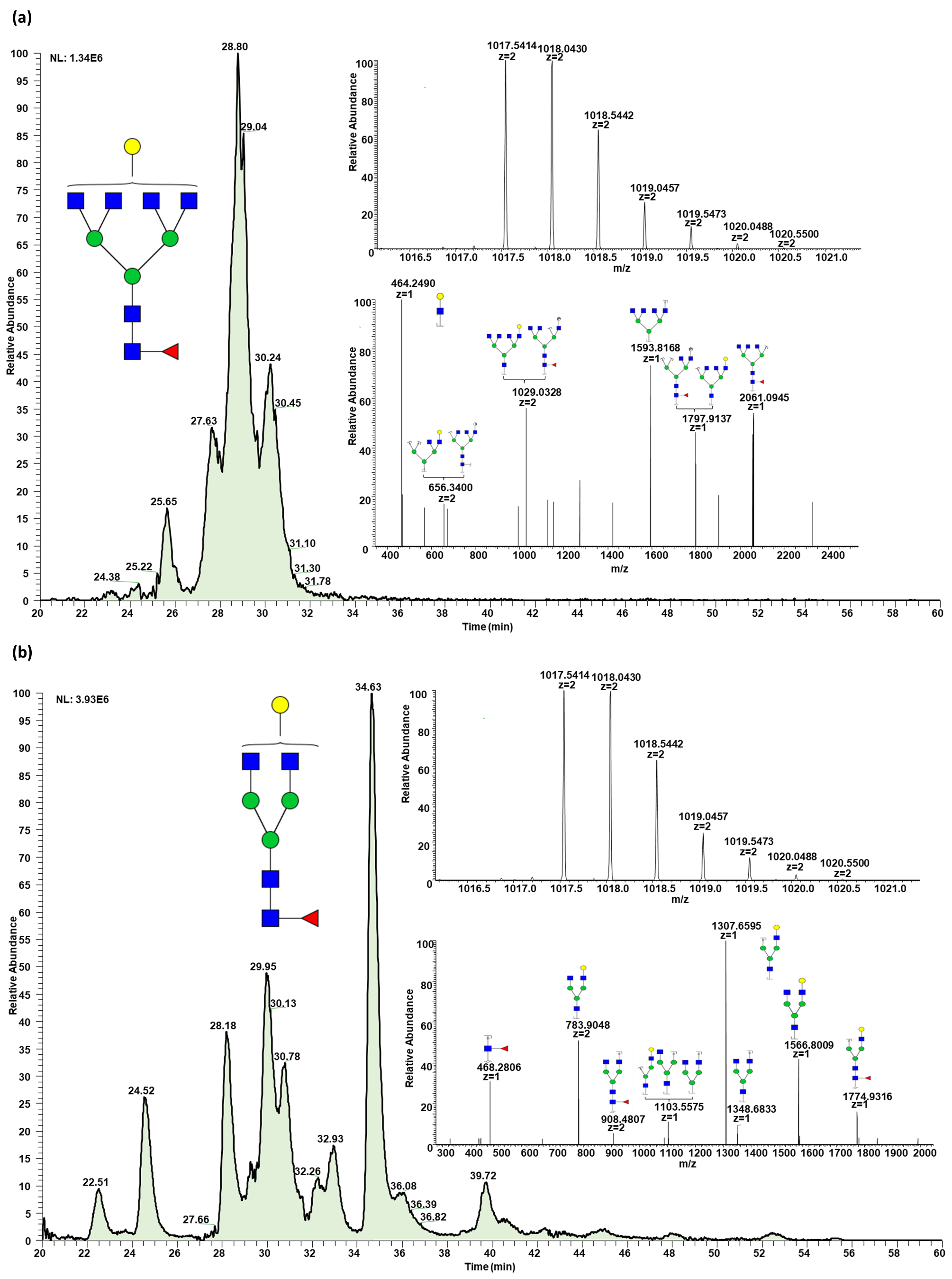
**

**Figure S-3:** Principal component analysis (PCA) with a confidence level of 95% of all identified *N*-glycans in the hippocampus section of the brain. Comparing Control vs GBH-Exposed cohorts; combined gender data (N=25).

**Figure S-4:** Distribution of all identified *N*-glycans by types between Control vs GBH-Exposed cohorts in (a) Male and (b) Female gender subgroups of the hippocampus section of the brain.

**
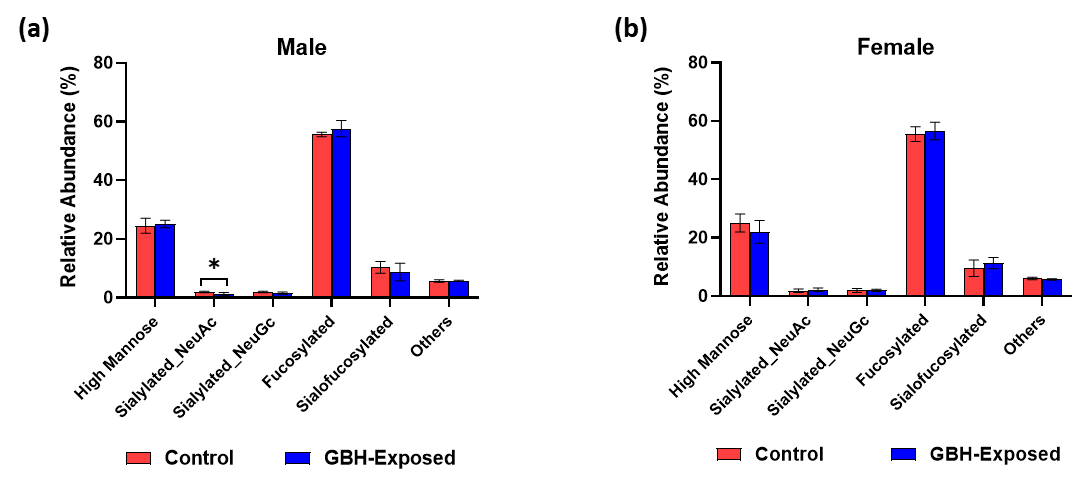
**

**Figure S-5:** Principal component analysis (PCA) with a confidence level of 95% of all identified *N*-glycans in the prefrontal cortex section of the brain. Comparing Control vs GBH-Exposed cohorts; combined gender data (N=25).

**
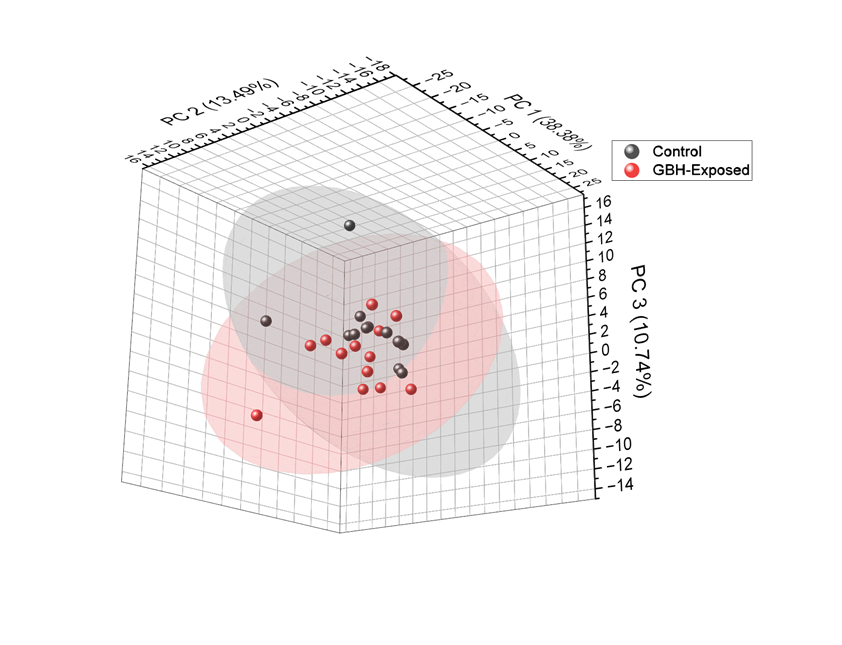
**

**Figure S-6:** Distribution of all identified *N*-glycans by types between Control vs GBH-Exposed cohorts in (a) Male and (b) Female gender subgroups of the prefrontal cortex section of the brain.

**
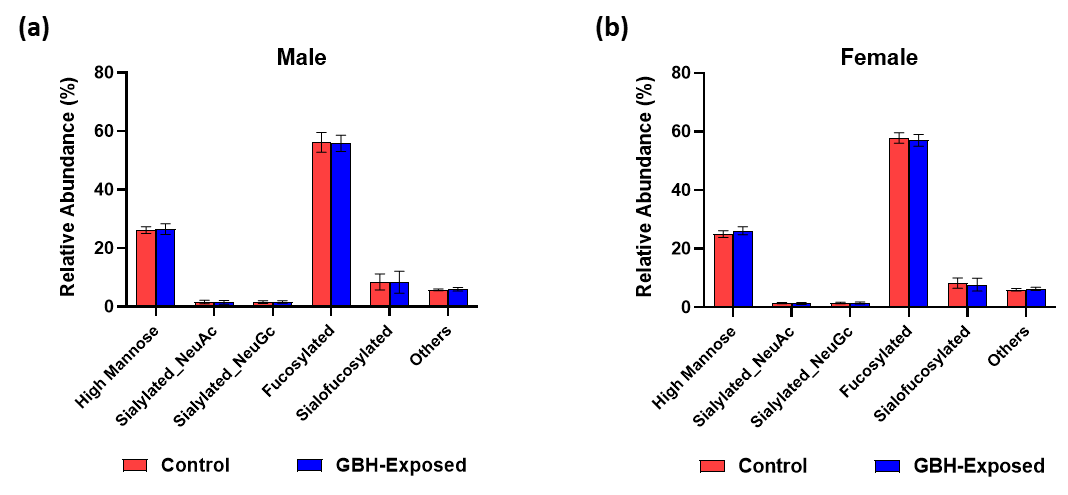
**

**Figure S-7:** Venn plot showing unique and overlapping significant *N*-glycans: in the gender subgroup data sets in (a) hippocampus section of the brain (b) prefrontal cortex section of the brain, and in the gender subgroup data sets between the hippocampus *vs* prefrontal cortex (c) Male (d) Female.

**
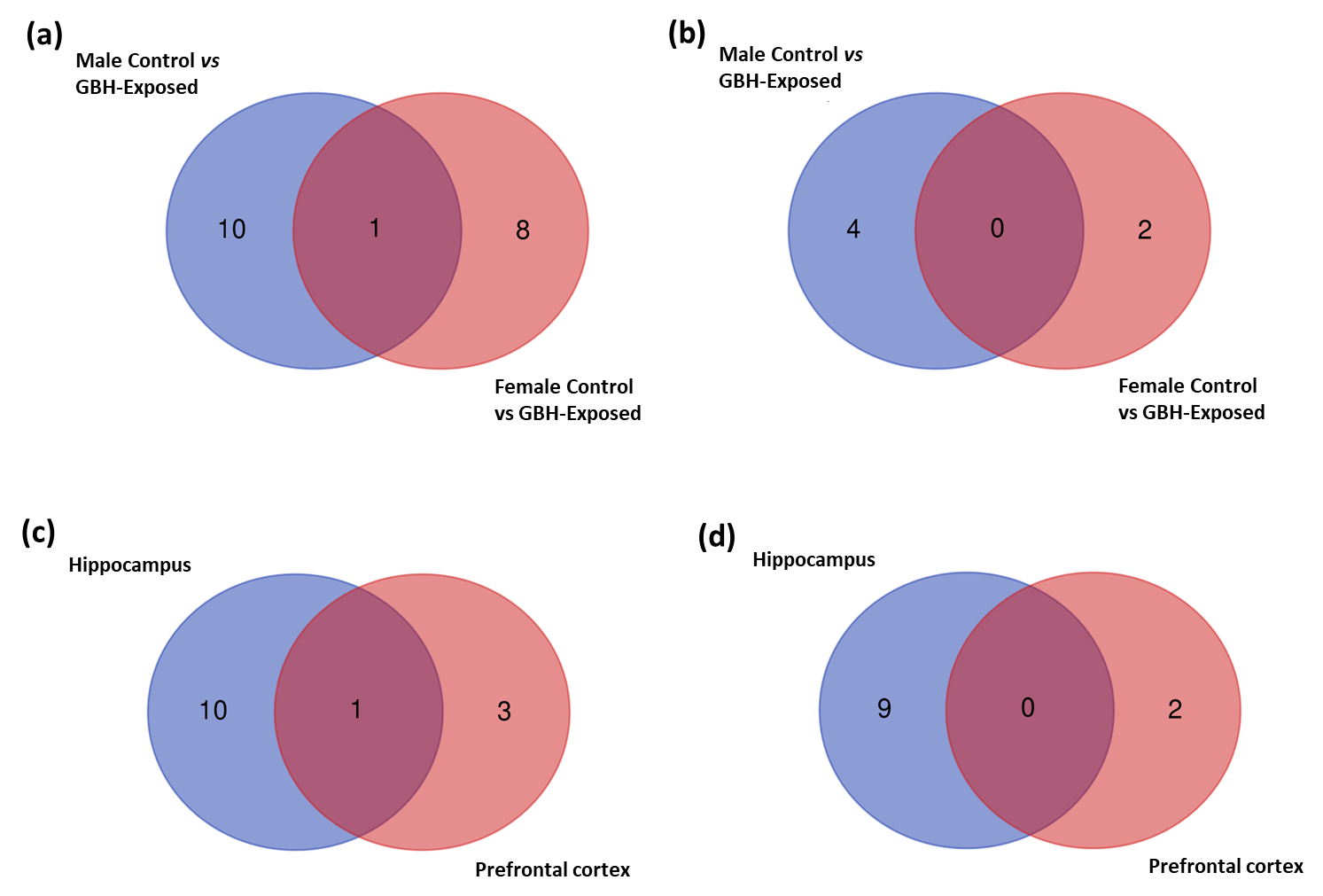
**

**SUPPLEMENTARY TABLES**

**Table S-1:** *N-*Glycans in the male gender rat subgroup of the hippocampus section of the brain validated by LC-PRM-MS, including precursor m/z, transition fragment ions, fold change (FC) and log2fc for the full scan and PRM validation.

| ***N-*Glycan Composition** | **Precursor (*m/z*)** | **Transition ion fragments** | **FC (Full Scan)** | **log2FC (Full Scan)** | **FC (PRM)** | **log2FC (PRM)** |
| --- | --- | --- | --- | --- | --- | --- |
| 45010 | 1213.1535 | 993.5083, 1348.6816, 1538.7632, 1726.868, 1797.9078 | 0.666 | -0.586 | 1.106 | 0.146 |
| 35010 | 1090.5903 | 1090.5304, 943.9731, 1307.6588, 1511.7531, 872.4182 | 0.712 | -0.490 | 0.941 | -0.0878 |
| 45020 | 1393.7403 | 825.4216, 1668.8316, 1913.9598, 2118.0583, 1247.6268 | 0.601 | -0.734 | 0.683 | -0.549 |
| 56200 | 1431.2689 | 1930.9691, 1198.1099, 1742.8589, 2399.2542, 2206.108 | 0.591 | -0.758 | 0.462 | -1.114 |
| 29000 | 1195.6399 | 1968.0121, 1763.9079, 1605.8251, 800.667, 1398.7544 | 1.412 | 0.497 | 1.514 | 0.598 |
| 54000 | 1053.0799 | 1552.7834, 906.4669, 1348.6838, 923.4571, 1846.9656 | 1.107 | 0.147 | 1.478 | 0.564 |
| 45110 | 1300.1981 | 1668.8287, 1756.8784, 1552.7821, 1307.655, 825.421 | 0.655 | -0.611 | 0.785 | -0.350 |

**Table S-2:** Statistically significant N-glycans between control relative to GBH-Exposed cohort in the male gender subgroup for hippocampus with their p-values, adjusted p-value, fold change (FC), and expression level.

| ***N-*Glycan Composition** | ***N-*Glycan Structure** | **p-value** | **Adjusted p-value** | **fold change (FC)** | **Expression** |
| --- | --- | --- | --- | --- | --- |
| **57200** | **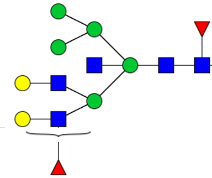** | **0.004** | **0.04** | **0.501** | **Downregulated** |
| **44101** | **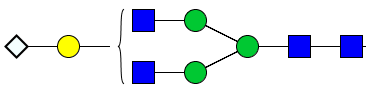** | **0.004** | **0.02** | **0.593** | **Downregulated** |
| **45010** | 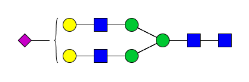 | **0.01** | **0.04** | **0.666** | **Downregulated** |
| **35010** | 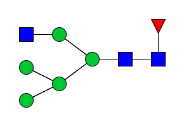 | **0.01** | **0.04** | **0.712** | **Downregulated** |
| **56210** | 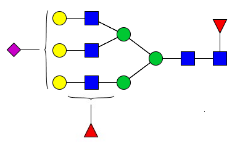 | **0.02** | **0.04** | **0.64** | **Downregulated** |
| **45020** | 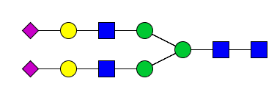 | **0.02** | **0.04** | **0.60** | **Downregulated** |
| **56200** | 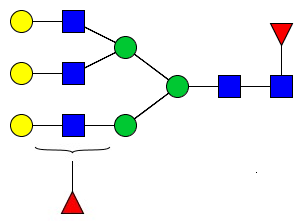 | **0.02** | **0.03** | **0.591** | **Downregulated** |
| **55110** | **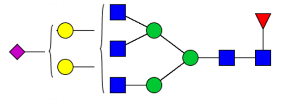** | **0.02** | **0.03** | **0.694** | **Downregulated** |
| ***N-*Glycan Composition** | ***N-*Glycan Structure** | **p-value** | **Adjusted p-value** | **fold change (FC)** | **Expression** |
| **29000** | 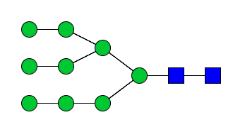 | **0.03** | **0.04** | **1.41** | **Upregulated** |
| **54000** | 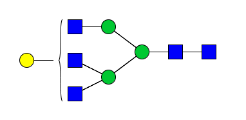 | **0.03** | **0.04** | **1.11** | **Upregulated** |
| **45110** | 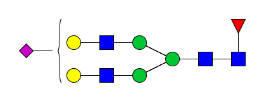 | **0.03** | **0.03** | **0.655** | **Downregulated** |

**Table S-3:** Statistically significant N-glycans between control relative to GBH-Exposed cohort in the female gender subgroup for hippocampus with their p-values, adjusted p-value, fold change (FC), and expression level. The N-glycan composition is HexNAc, Hex, Fucose, NeuAc and NeuGc.

| ***N-*Glycan Composition** | ***N-*Glycan Structure** | **p-value** | **Adjusted p-value** | **fold change (FC)** | **Expression** |
| --- | --- | --- | --- | --- | --- |
| **75210** | **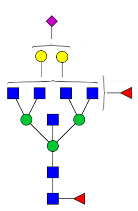** | **0.004** | **0.04** | **1.676** | **Upregulated** |
| **65300** | 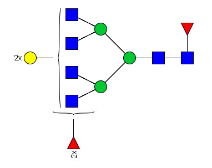 | **0.006** | **0.04** | **1.262** | **Upregulated** |
| **45210** | 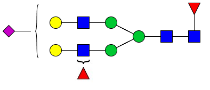 | **0.01** | **0.04** | **1.318** | **Upregulated** |
| **53000** | 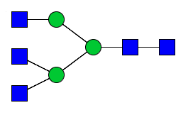 | **0.01** | **0.04** | **0.902** | **Downregulated** |
| **67230** | 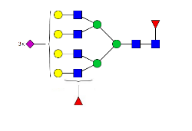 | **0.03** | **0.04** | **1.782** | **Upregulated** |
| **54000** | 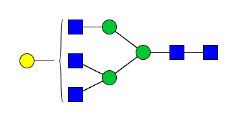 | **0.03** | **0.04** | **0.837** | **Downregulated** |
| **67220** | 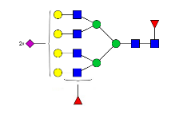 | **0.03** | **0.04** | **1.496** | **Upregulated** |
| **66400** | 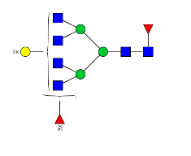 | **0.04** | **0.04** | **0.632** | **Downregulated** |
| **67340** | 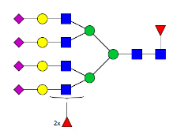 | **0.04** | **0.04** | **1.676** | **Upregulated** |

**Table S-4:** Statistically significant N-glycans between control relative to GBH-Exposed cohort in the male gender subgroup for prefrontal cortex with their p-values, adjusted p-value, fold change (FC), and expression level. The N-glycan composition is HexNAc, Hex, Fucose, NeuAc and NeuGc.

| ***N-*Glycan Composition** | ***N-*Glycan Structure** | **p-value** | **Adjusted p-value** | **fold change (FC)** | **Expression** |
| --- | --- | --- | --- | --- | --- |
| **64100** | 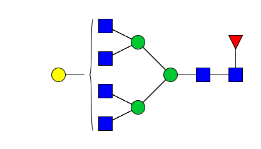 | **0.01** | **0.04** | **0.778** | **Downregulated** |
| **44100** | 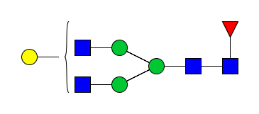 | **0.02** | **0.04** | **0.900** | **Downregulated** |
| **57200** | **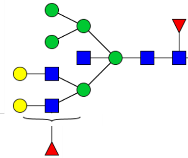** | **0.02** | **0.03** | **0.648** | **Downregulated** |
| **23000** | 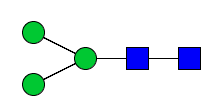 | **0.03** | **0.03** | **1.057** | **Upregulated** |

**Table S-5:** Statistically significant N-glycans between control relative to GBH-Exposed cohort in the female gender subgroup for prefrontal cortex with their p-values, adjusted p-value, fold change (FC), and expression level. The N-glycan composition is HexNAc, Hex, Fucose, NeuAc and NeuGc.

| ***N-*Glycan Composition** | ***N-*Glycan Structure** | **p-value** | **Adjusted p-value** | **fold change (FC)** | **Expression** |
| --- | --- | --- | --- | --- | --- |
| **26000** | 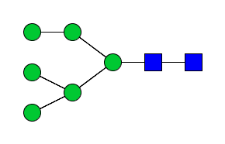 | **0.01** | **0.02** | **1.089** | **Upregulated** |
| **45110** | 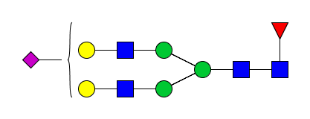 | **0.04** | **0.04** | **0.826** | **Downregulated** |

**Table S-6:** Significant *N-*Glycans common and unique between the gender cohorts of the hippocampus section of the brain.

**Table S-7:** Significant *N-*Glycans common and unique between the gender cohorts of the prefrontal cortex section of the brain.

|  | **NAMES** | **TOTAL** | **GLYCANS** |
| --- | --- | --- | --- |
| **COMMON** | **Male & Female Control *vs* GBH-Exposed (Prefrontal cortex)** | **0** |  |
| **UNIQUE** | **Male Control *vs* GBH-Exposed (Prefrontal Cortex)** | **4** | **23000** |
|  |  |  | **57200** |
|  |  |  | **64100** |
|  |  |  | **44100** |
| **UNIQUE** | **Female Control *vs* GBH-Exposed (Prefrontal Cortex)** | **2** | **45110** |
|  |  |  | **26000** |

**Table S-8:** Significant *N-*Glycans common and unique in the combined group comparing the hippocampus section to the prefrontal cortex of the brain.

|  | **NAMES** | **TOTAL** | **GLYCANS** |
| --- | --- | --- | --- |
| **COMMON** | **Hippocampus *vs* Prefrontal Cortex** | **0** |  |
| **UNIQUE** | **Hippocampus** | **2** | **63100** |
|  |  |  | **54101** |
| **UNIQUE** | **Prefrontal Cortex** | **3** | **67300** |
|  |  |  | **57200** |
|  |  |  | **45110** |

**Table S-9:** Significant *N-*Glycans common and unique in the male gender rat group comparing the hippocampus section to the prefrontal cortex of the brain.

|  | **NAMES** | **TOTAL** | **GLYCANS** |
| --- | --- | --- | --- |
| **COMMON** | **Hippocampus *vs* Prefrontal Cortex** | **1** | **57200** |
| **UNIQUE** | **Hippocampus** | **10** | **55110** |
|  |  |  | **45020** |
|  |  |  | **44101** |
|  |  |  | **45010** |
|  |  |  | **45110** |
|  |  |  | **35010** |
|  |  |  | **56210** |
|  |  |  | **29000** |
|  |  |  | **54000** |
|  |  |  | **56200** |
| **UNIQUE** | **Prefrontal Cortex** | **3** | **44100** |
|  |  |  | **23000** |
|  |  |  | **64100** |

**Table S-10:** Significant *N-*Glycans common and unique in the female gender rat group comparing the hippocampus section to the prefrontal cortex of the brain.

|  | **NAMES** | **TOTAL** | **GLYCANS** |
| --- | --- | --- | --- |
| **COMMON** | **Hippocampus *vs* Prefrontal Cortex** | **0** |  |
| **UNIQUE** | **Hippocampus** | **9** | **67220** |
|  |  |  | **53000** |
|  |  |  | **67230** |
|  |  |  | **75210** |
|  |  |  | **65300** |
|  |  |  | **45210** |
|  |  |  | **67340** |
|  |  |  | **66400** |
|  |  |  | **54000** |
| **UNIQUE** | **Prefrontal Cortex** | **2** | **45110** |
|  |  |  | **26000** |
